# Permutation inference for the general linear model

**DOI:** 10.1016/j.neuroimage.2014.01.060

**Published:** 2014-05-15

**Authors:** Anderson M. Winkler, Gerard R. Ridgway, Matthew A. Webster, Stephen M. Smith, Thomas E. Nichols

**Affiliations:** a Oxford Centre for Functional MRI of the Brain, University of Oxford, Oxford, UK; b Global Imaging Unit, GlaxoSmithKline, London, UK; c Department of Psychiatry, Yale University School of Medicine, New Haven, CT, USA; d Wellcome Trust Centre for Neuroimaging, UCL Institute of Neurology, London, UK; e Department of Statistics & Warwick Manufacturing Group, University of Warwick, Coventry, UK

**Keywords:** Permutation inference, Multiple regression, General linear model, Randomise

## Abstract

Permutation methods can provide exact control of false positives and allow the use of non-standard statistics, making only weak assumptions about the data. With the availability of fast and inexpensive computing, their main limitation would be some lack of flexibility to work with arbitrary experimental designs. In this paper we report on results on approximate permutation methods that are more flexible with respect to the experimental design and nuisance variables, and conduct detailed simulations to identify the best method for settings that are typical for imaging research scenarios. We present a generic framework for permutation inference for complex general linear models (glms) when the errors are exchangeable and/or have a symmetric distribution, and show that, even in the presence of nuisance effects, these permutation inferences are powerful while providing excellent control of false positives in a wide range of common and relevant imaging research scenarios. We also demonstrate how the inference on glm parameters, originally intended for independent data, can be used in certain special but useful cases in which independence is violated. Detailed examples of common neuroimaging applications are provided, as well as a complete algorithm – the “randomise” algorithm – for permutation inference with the glm.

## Highlights

• Permutation for the GLM in the presence of nuisance or non-independence.
• A generalised statistic that performs well even under heteroscedasticity.
• Permutation and/or sign-flipping, exchangeability blocks and variance groups.
• The “randomise” algorithm, as well as various practical examples.

## Introduction

The field of neuroimaging has continuously expanded to encompass an ever growing variety of experimental methods, each of them producing images that have different physical and biological properties, as well as different information content. Despite the variety, most of the strategies for statistical analysis can be formulated as a general linear model (glm) (Christensen, 2002; Scheffé, 1959; Searle, 1971). The common strategy is to construct a plausible explanatory model for the observed data, estimate the parameters of this model, and compute a suitable statistic for hypothesis testing on some or all of these parameters. The rejection or acceptance of a hypothesis depends on the probability of finding, due to chance alone, a statistic at least as extreme as the one observed. If the distribution of the statistic under the null hypothesis is known, such probability can be ascertained directly. In order to be valid, these *parametric tests* rely on a number of assumptions under which such distribution arises and can be recovered asymptotically. Strategies that may be used when these assumptions are not guaranteed to be met include the use of *non-parametric tests*.

*Permutation tests* are a class of non-parametric methods. They were pioneered by Fisher (1935a) and Pitman (1937a,b, 1938). Fisher demonstrated that the null hypothesis could be tested simply by observing, after permuting observations, how often the difference between means would exceed the difference found without permutation, and that for such test, no normality would be required. Pitman provided the first complete mathematical framework for permutation methods, although similar ideas, based on actually repeating an experiment many times with the experimental conditions being permuted, can be found even earlier (Peirce and Jastrow, 1884). Important theoretical and practical advances have been ongoing in the past decades (Edgington, 1995; Good, 2002, 2005; Kempthorne, 1955; Lehmann and Stein, 1949; Pearson, 1937; Pesarin and Salmaso, 2010; Scheffé, 1943; Westfall and Troendle, 2008), and usage only became practical after the availability sufficient computing power (Efron, 1979).

In neuroimaging, permutation methods were first proposed by Blair et al. (1994) for electroencephalography, and later by Holmes et al. (1996) for positron-emission tomography, with the objective of allowing inferences while taking into account the multiplicity of tests. These early permutation approaches already accounted for the spatial smoothness of the image data. Arndt et al. (1996) proposed a permutation scheme for testing the omnibus hypothesis of whether two sets of images would differ. Structural magnetic resonance imaging (mri) data were considered by Bullmore et al. (1999), who developed methods for omnibus, voxel and cluster-mass inference, controlling the expected number of false positives.

Single subject experiments from functional magnetic resonance imaging (fmri) presents a challenge to permutation methods, as serial autocorrelation in the time series violates the fundamental assumption needed for permutation, that of exchangeability (discussed below). Even though some early work did not fully account for autocorrelation (Belmonte and Yurgelun-Todd, 2001), other methods that accommodated the temporally correlated nature of the fmri signal and noise were developed (Brammer et al., 1997; Breakspear et al., 2004; Bullmore et al., 1996, 2001; Laird et al., 2004; Locascio et al., 1997). Some of these methods use a single reference distribution constructed by pooling permutation statistics over space from a small number of random permutations, under the (untenable and often invalid) assumption of spatial homogeneity of distributions.

Nichols and Holmes (2002) provided a practical description of permutation methods for pet and multi-subject fmri studies, but noted the challenges posed by nuisance variables. Permutation inference is grounded on *exchangeability* under the null hypothesis, that data can be permuted (exchanged) without affecting its joint distribution. However, if a nuisance effect is present in the model, the data cannot be considered exchangeable even under the null hypothesis. For example, if one wanted to test for sex differences while controlling for the linear effect of age, the null hypothesis is “male mean equals female mean”, while allowing age differences; the problem is that, even when there is no sex effect, a possible age effect may be present, e.g., younger and older individuals being different, then the data are not directly exchangeable under this null hypothesis. Another case where this arises is in factorial experiments, where one factor is to be tested in the presence of another, or where their interaction is to be tested in the presence of main effects of either or both. Although permutation strategies for factorial experiments in neuroimaging were considered by Suckling and Bullmore (2004), a more complete general framework to account for nuisance variables is still missing.

In this paper we review the statistical literature for the glm with arbitrary designs and contrasts, emphasising useful aspects, yet that have not been considered for neuroimaging, unify this diverse set of results into a single permutation strategy and a single generalised statistic, present implementation strategies for efficient computation and provide a complete algorithm, conduct detailed simulations and evaluations in various settings, and identify certain methods that generally outperforms others. We will not consider intrasubject (timeseries) fmri data, focusing instead on modelling data with independent observations or sets of non-independent observations from independent subjects. We give examples of applications to common designs and discuss how these methods, originally intended for independent data, can in special cases be extended, e.g., to repeated measurements and longitudinal designs.

## Theory

### Model and notation

At each spatial point (voxel, vertex or face) of an image representation of the brain, a general linear model (Searle, 1971) can be formulated and expressed as:

(1) Y=Mψ+ϵ

where **Y** is the *N* × 1 vector of observed data,^1^ **M** is the full-rank *N* × r design matrix that includes all effects of interest as well as all modelled nuisance effects, *ψ* is the *r* × 1 vector of *r* regression coefficients, and *ϵ* is the *N* × 1 vector of random errors. In permutation tests, the errors are not assumed to follow a normal distribution, although some distributional assumptions are needed, as detailed below. Estimates for the regression coefficients can be computed as $\hat{\psi }=M^{+}Y$, where the superscript (^+^) denotes the Moore–Penrose pseudo-inverse. Our interest is to test the null hypothesis that an arbitrary combination (contrast) of some or all of these parameters is equal to zero, i.e., H_0_ : **C**′*ψ* = 0, where **C** is a *r* × *s* full-rank matrix of *s* contrasts, 1 ≤ *s* ≤ *r*.

For the discussion that follows, it is useful to consider a transformation of the model in Eq. (1) into a partitioned one:

(2) Y=Xβ+Zγ+ϵ

where **X** is the matrix with regressors of interest, **Z** is the matrix with nuisance regressors, and *β* and *γ* are the vectors of regression coefficients. Even though such partitioning is not unique, it can be defined in terms of the contrast **C** in a way that inference on *β* is equivalent to inference on **C**′*ψ*, as described in Appendix A. As the partitioning depends on **C**, if more than one contrast is tested, **X** and **Z** change for each of them.

As the models expressed in Eqs. (1) and (2) are equivalent, their residuals are the same and can be obtained as $\hat{\epsilon }=R_{M}Y$, where **R**_**M**_ = **I** − **H**_**M**_ is the residual-forming matrix, **H**_**M**_ = **MM**^+^ is the projection (“hat”) matrix, and **I** is the *N* × *N* identity matrix. The residuals due to the nuisance alone are ${\hat{\epsilon }}_{Z}=R_{Z}Y$, where **R**_**Z**_ = **I** − **H**_**Z**_, and **H**_**Z**_ = **ZZ**^+^. For permutation methods, an important detail of the linear model is the non-independence of residuals, even when errors ϵ are independent and have constant variance, a fact that contributes to render these methods approximately exact. For example, in that setting $E\left(\mathrm{Var}\left({\hat{\epsilon }}_{Z}\right)\right)=R_{Z}\neq I$. The commonly used *F* statistic can be computed as (Christensen, 2002):

(3) $$\begin{array}{c} F=\frac{{\hat{\psi }}^{\prime }C{\left(C^{\prime }{\left(M^{\prime }M\right)}^{-1}C\right)}^{-1}C^{\prime }\hat{\psi }}{\mathrm{rank}\left(C\right)}/\frac{{\hat{\epsilon }}^{\prime }\hat{\epsilon }}{N-\mathrm{rank}\left(M\right)}\\ =\frac{{\hat{\beta }}^{\prime }\left(X^{\prime }X\right)\hat{\beta }}{\mathrm{rank}\left(C\right)}/\frac{{\hat{\epsilon }}^{\prime }\hat{\epsilon }}{N-\mathrm{rank}\left(X\right)-\mathrm{rank}\left(Z\right)}. \end{array}$$

When $\mathrm{rank}\left(C\right)=1,\hat{\beta }$ is a scalar and the Student's *t* statistic can be expressed as a function of *F* as $t=\mathrm{sign}\left(\hat{\beta }\right)\sqrt{F}$.

#### Choice of the statistic

In non-parametric settings we are not constrained to the *F* or *t* statistics and, in principle, any statistic where large values reflect evidence against the null hypothesis could be used. This includes regression coefficients or descriptive statistics, such as differences between medians, trimmed means or ranks of observations (Ernst, 2004). However, the statistic should be chosen such that it does not depend on the scale of measurement or on any unknown parameter. The regression coefficients, for instance, whose variance depends both on the error variance and on the collinearity of that regressor with the others, are not in practice a good choice, as certain permutation schemes alter the collinearity among regressors (Kennedy and Cade, 1996). Specifically with respect to brain imaging, the correction for multiple testing (discussed later) requires that the statistic has a distribution that is spatially homogeneous, something that regression coefficients cannot provide. In parametric settings, statistics that are independent of any unknown parameters are called *pivotal statistics*. Statistics that are pivotal or asymptotically pivotal are appropriate and facilitate the equivalence of the tests across the brain, and their advantages are well established for related non-parametric methods (Hall and Wilson, 1991; Westfall and Young, 1993). Examples of such pivotal statistics include the Student's *t*, the *F* ratio, the Pearson's correlation coefficient (often known as *r*), the coefficient of determination (*R*^2^), as well as most other statistics used to construct confidence intervals and to compute p-values in parametric tests. We will return to the matter of pivotality when discussing exchangeability blocks, and the choice of an appropriate statistic for these cases.

#### p-Values

Regardless of the choice of the test statistic, p-values offer a common measure of evidence against the null hypothesis. For a certain test statistic *T*, which can be any of those discussed above, and a particular observed value *T*_0_ of this statistic after the experiment has been conducted, the p-value is the probability of observing, by chance, a test statistic equal or larger than the one computed with the observed values, i.e., *P*(*T* ≥ *T*_0_|H_0_). Although here we only consider one-sided tests, where evidence against H_0_ corresponds to larger values of *T*_0_, two-sided or negative-valued tests and their p-values can be similarly defined. In parametric settings, under a number of assumptions, the p-values can be obtained by referring to the theoretical distribution of the chosen statistic (such as the *F* distribution), either through a known formula, or using tabulated values. In non-parametric settings, these assumptions are avoided. Instead, the data are randomly shuffled, many times, in a manner consistent with the null hypothesis. The model is fitted repeatedly once for every shuffle, and for each fit a new realisation of the statistic, *T*_*j*_^⁎^, is computed, being *j* a permutation index. An empirical distribution of *T*^⁎^ under the null hypothesis is constructed, and from this null distribution a p-value is computed as $\frac{1}{J}\sum_{j}\;I\left(T_{j}^{⁎}\geq T_{0}\right)$, where *J* is the number of shufflings performed, and *I*(∙) is the indicator function. From this it can be seen that the non-parametric p-values are discrete, with each possible p-value being a multiple of 1/*J*. It is important to note that the permutation distribution should include the observed statistic without permutation (Edgington, 1969; Phipson and Smyth, 2010), and thus the smallest possible p-value is 1/*J*, not zero.

### Permutations and exchangeability

**Fig. 1:**
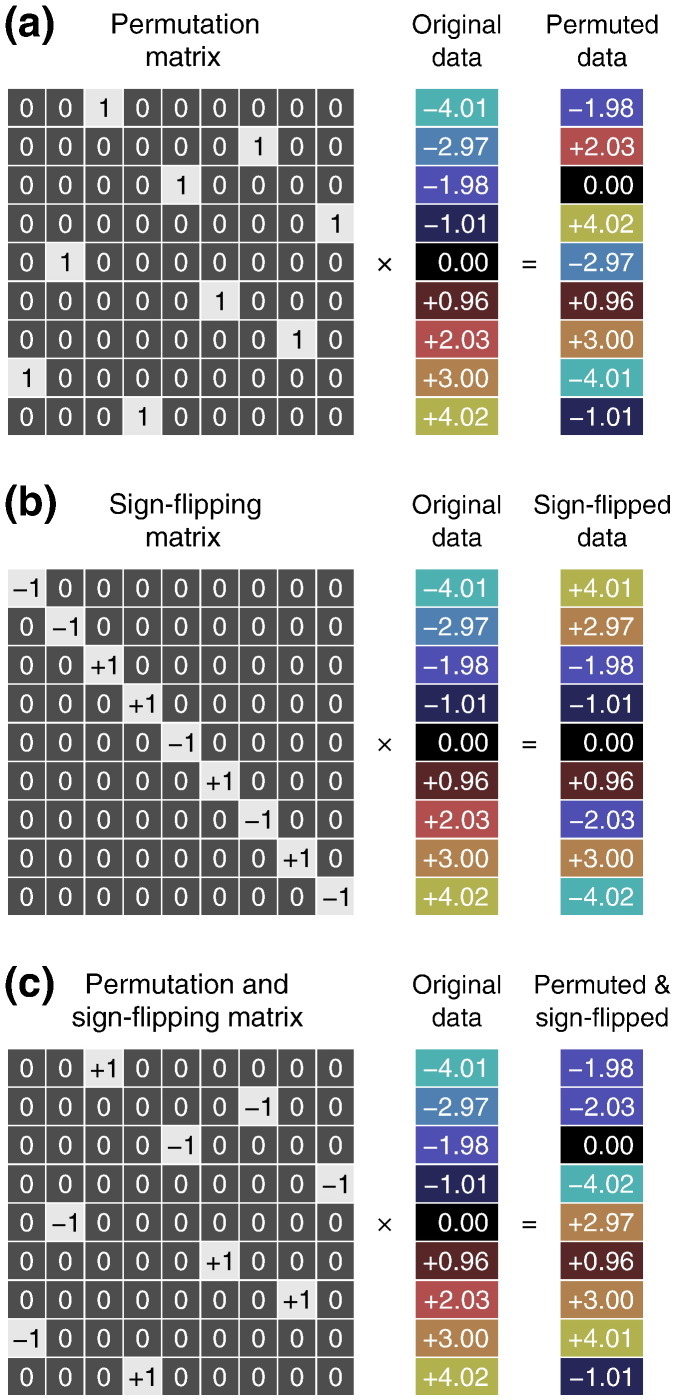
Examples of a permutation matrix (a), of a sign flipping matrix (b), and of a matrix that does permutation and sign flipping (c). Pre-multiplication by a permutation matrix shuffles the order of the data, whereas by a sign flipping matrix changes the sign of a random subset of data points.

Perhaps the most important aspect of permutation tests is the manner in which data are shuffled under the null hypothesis. It is the null hypothesis, together with assumptions about exchangeability, which determines the permutation strategy. Let the *j*-th permutation be expressed by **P***_j_*, a *N* × *N* permutation matrix, a matrix that has all elements being either 0 or 1, each row and column having exactly one 1 (Fig. 1a). Pre-multiplication of a matrix by **P***_j_* permutes its rows. We denote $P=\left\{P_{j}\right\}$ the set of all permutation matrices under consideration, indexed by the subscript *j*. We similarly define a sign flipping matrix **S***_j_*, a *N* × *N* diagonal matrix whose non-zero elements consist only of + 1 or − 1 (Fig. 1b). Pre-multiplication of a matrix by **S***_j_* implements a set of sign flips for each row. Likewise, $S=\left\{S_{j}\right\}$ denotes the set of all sign flipping matrices under consideration. We consider also both schemes together, where $B_{j}=P_{j^{\prime }}S_{j^{\prime \prime }}$ implements sign flips followed by permutation; the set of all possible such transformations is denoted as B = {**B**_*j*_}. Throughout the paper, we use generic terms as *shuffling* or *rearrangement* whenever the distinction between permutation, sign flipping or combined permutation with sign flipping is not pertinent. Finally, let ${\hat{\beta }}_{j}^{*}$ and *T*_*j*_^⁎^, respectively, be the estimated regression coefficients and the computed statistic for the shuffling *j*.

The essential assumption of permutation methods is that, for a given set of variables, *their joint probability distribution does not change if they are rearranged*. This can be expressed in terms of exchangeable errors or independent and symmetric errors, each of these weakening different assumptions when compared to parametric methods.

*Exchangeable errors* (ee) is the traditional permutation requirement (Good, 2005). The formal statement is that, for any permutation $P_{j}\in P$, $\epsilon^{\underline{\underline{d}}}P_{j}\epsilon$, where the symbol ${}^{\underline{\underline{d}}}$ denotes equality of distributions. In other words, the errors are considered exchangeable if their joint distribution is invariant with respect to permutation. Exchangeability is similar to, yet more general than, independence, as exchangeable errors can have all-equal and homogeneous dependence. Relative to the common parametric assumptions of independent, normally and identically distributed (iid) errors, ee relaxes two aspects. First, normality is no longer assumed, although identical distributions are required. Second, the independence assumption is weakened slightly to allow exchangeability when the observations are not independent, but their joint distribution is maintained after permutation. While exchangeability is a general condition that applies to any distribution, we note that the multivariate normal distribution is indeed exchangeable if all off-diagonal elements of the covariance matrix are identical to each other (not necessarily equal to zero) and all the diagonal elements are also identical to each other. In parametric settings, such dependence structure is often referred to as *compound symmetry*.

*Independent and symmetric errors* (ise) can be considered for measurements that arise, for instance, from differences between two groups if the variances are not assumed to be the same. The formal statement for permutation under ise is that for any sign flipping matrix $S_{j}\in S,\epsilon^{\underline{\underline{d}}}S_{j}\epsilon$, that is, the joint distribution of the error terms is invariant with respect to sign flipping. Relative to the parametric assumptions of independent, normally and identically distributed errors, ise relaxes normality, although symmetry (i.e., non-skewness) of distributions is required. Independence is also required to allow sign flipping of one observation without perturbing others.

The choice between ee and ise depends on the knowledge of, or assumptions about, the error terms. Although the ee does not require symmetry for the distribution of the error terms, it requires that the variances and covariances of the error terms are all equal, or have a structure that is compatible with the definition of exchangeability blocks (discussed below). While the ise assumption has yet more stringent requirements, if both ee and ise are plausible and available for a given model, permutations and sign flippings can be performed together, increasing the number of possible rearrangements, a feature particularly useful for studies with small sample sizes. The formal statement for shuffling under both ee and ise is that, as with the previous cases, for any matrix $B_{j}\in \beta ,\epsilon^{\underline{\underline{d}}}B_{j}\epsilon$, that is, the joint distribution of the error terms remains unchanged under both permutation and sign flipping. A summary of the properties discussed thus far and some benefits of permutation methods are shown in Table 1.

There are yet other important aspects related to exchangeability. The experimental design may dictate blocks of observations that are jointly exchangeable, allowing data to be permuted within block or, alternatively, that the blocks may themselves be exchangeable as a whole. This is the case, for instance, for designs that involve multiple observations from each subject. While permutation methods generally do not easily deal with non-independent data, the definition of these *exchangeability blocks* (eb) allows these special cases of well structured dependence to be accommodated. Even though the ebs determine how the data shufflings are performed, they should not be confused with *variance groups* (vg), i.e., groups of observations that are known or assumed to have similar variances, which can be pooled for estimation and computation of the statistic. Variance groups need to be compatible with, yet not necessarily identical to, the exchangeability blocks, as discussed in Restricted exchangeability.

#### Unrestricted exchangeability

In the absence of nuisance variables, the model reduces to **Y** = **X***β* + ϵ, and under the null hypothesis H_0_ : *β* = 0, the data are pure error, **Y** = ϵ. Thus the ee or ise assumptions on the *error* (presented above) justify freely permuting or sign flipping the *data* under H_0_. It is equivalent, however, to alter the design instead of the data. For example, for a nuisance-free design,

(4) $$\mathrm{PY}=X\beta +\epsilon \Leftrightarrow Y=P^{\prime }X\beta +P^{\prime }\epsilon$$

since permutation matrices **P** are orthogonal; the same holds for sign flipping matrices **S**. This is an important computational consideration as altering the design is much less burdensome than altering the image data. The errors ϵ are not observed and thus never directly altered; going forward we will suppress any notation indicating permutation or sign flipping of the errors.

In the presence of nuisance variables (Eq. 2), however, the problem is more complex. If the nuisance coefficients *γ* were somehow known, an exact permutation test would be available:

(5) Y−Zγ=PXβ+ϵ.

The perfectly adjusted data **Y** − **Z***γ* are then pure error under H_0_ and inference could proceed as above. In practice, the nuisance coefficients have to be estimated and the adjusted data will not behave as ϵ. An obvious solution would be to use the nuisance-only residuals ${\hat{\epsilon }}_{Z}$ as the adjusted data. However, as noted above, residuals induce dependence and any ee or ise assumptions on ϵ will not be conveyed to ${\hat{\epsilon }}_{Z}$.

A number of approaches have been proposed to produce approximate p-values in these cases (Beaton, 1978; Brown and Maritz, 1982; Draper and Stoneman, 1966; Edgington, 1995; Freedman and Lane, 1983; Gail et al., 1988; Huh and Jhun, 2001; Jung et al., 2006; Kennedy, 1995; Kherad-Pajouh and Renaud, 2010; Levin and Robbins, 1983; Manly, 2007; Oja, 1987; Still and White, 1981; ter Braak, 1992; Welch, 1990). We present these methods in a common notation with detailed annotation in Table 2. While a number of authors have made comparisons between some of these methods (Anderson and Legendre, 1999; Anderson and Robinson, 2001; Anderson and ter Braak, 2003; Dekker et al., 2007; Gonzalez and Manly, 1998; Kennedy, 1995; Kennedy and Cade, 1996; Nichols et al., 2008; O'Gorman, 2005; Ridgway, 2009), they often only approached particular cases, did not consider the possibility of permutation of blocks of observations, did not use full matrix notation as more common in neuroimaging literature, and often did not consider implementation complexities due to the large size of imaging datasets. In this section we focus on the Freedman–Lane and the Smith methods, which, as we show in Permutation strategies, produce the best results in terms of control over error rates and power.

The *Freedman–Lane procedure* (Freedman and Lane, 1983) can be performed through the following steps:

1. Regress **Y** against the full model that contains both the effects of interest and the nuisance variables, i.e. **Y** = **X***β* + **Z***γ* + ϵ. Use the estimated parameters $\hat{\beta }$ to compute the statistic of interest, and call this statistic *T*_0_.
2. Regress **Y** against a reduced model that contains only the nuisance effects, i.e. **Y** = **Z***γ* + ϵ_**Z**_, obtaining estimated parameters $\hat{\gamma }$ and estimated residuals ${\hat{\epsilon }}_{Z}$.
3. Compute a set of permuted data **Y**_*j*_^∗^. This is done by pre-multiplying the residuals from the reduced model produced in the previous step, ${\hat{\epsilon }}_{Z}$, by a permutation matrix, **P***_j_*, then adding back the estimated nuisance effects, i.e. $Y_{j}^{*}=P_{j}{\hat{\epsilon }}_{Z}+Z\hat{\gamma }$.
4. Regress the permuted data **Y**_*j*_^∗^ against the full model, i.e. **Y**_*j*_^∗^ = **X***β* + **Z***γ* + ϵ, and use the estimated ${\hat{\beta }}_{j}^{*}$ to compute the statistic of interest. Call this statistic *T*_*j*_^∗^.
5. Repeat Steps 2–4 many times to build the reference distribution of *T*^⁎^ under the null hypothesis.
6. Count how many times *T*_*j*_^∗^ was found to be equal to or larger than *T*_0_, and divide the count by the number of permutations; the result is the p-value.

For Steps 2 and 3, it is not necessary to actually fit the reduced model at each point in the image. The permuted dataset can equivalently be obtained as **Y**_*j*_^∗^ = (**P**_*j*_**R**_**Z**_ + **H**_**Z**_)**Y**, which is particularly efficient for neuroimaging applications in the typical case of a single design matrix for all image points, as the term **P**_*j*_**R**_**Z**_ + **H**_**Z**_ is then constant throughout the image and so, needs to be computed just once. Moreover, the addition of nuisance variables back in Step 3 is not strictly necessary, and the model can be expressed simply as **P**_*j*_**R**_**Z**_**Y** = **X***β* + **Z***γ* + ϵ, implying that the permutations can actually be performed just by permuting the rows of the residual-forming matrix **R_Z_**. The Freedman–Lane strategy is the one used in the randomise algorithm, discussed in Appendix B.

The rationale for this permutation method is that, if the null hypothesis is true, then *β* = 0, and so the residuals from the reduced model with only nuisance variables, ϵ_***Z***_, should not be different than the residuals from the full model, ϵ, and can, therefore, be used to create the reference distribution from which p-values can be obtained.

The *Smith procedure* consists of orthogonalising the regressors of interest with respect to the nuisance variables. This is done by pre-multiplication of **X** by the residual forming matrix due to **Z**, i.e., **R_Z_**, then permuting this orthogonalised version of the regressors of interest. The nuisance regressors remain in the model.^2^

For both the Freedman–Lane and the Smith procedures, if the errors are independent and symmetric (ise), the permutation matrices **P***_j_* can be replaced for sign flipping matrices **S***_j_*. If both ee and ise are considered appropriate, then permutation and sign flipping can be used concomitantly.

#### Restricted exchangeability

Some experimental designs involve multiple observations from each subject, or the subjects may come from groups that may possess characteristics that may render their distributions not perfectly comparable. Both situations violate exchangeability. However, when the dependence between observations has a block structure, this structure can be taken into account when permuting the model, restricting the set of all otherwise possible permutations to only those that respect the relationship between observations (Pesarin, 2001); observations that are exchangeable only in some subsets of all possible permutations are said *weakly exchangeable* (Good, 2002). The ee and ise assumptions are then asserted at the level of these exchangeability blocks, rather than for each observation individually. The experimental hypothesis and the study design determine how the ebs should be formed and how the permutation or sign flipping matrices should be constructed. Except Huh–Jhun, the other methods in Table 2 can be applied at the block level as in the unrestricted case.

##### Within-block exchangeability

**Fig. 2:**
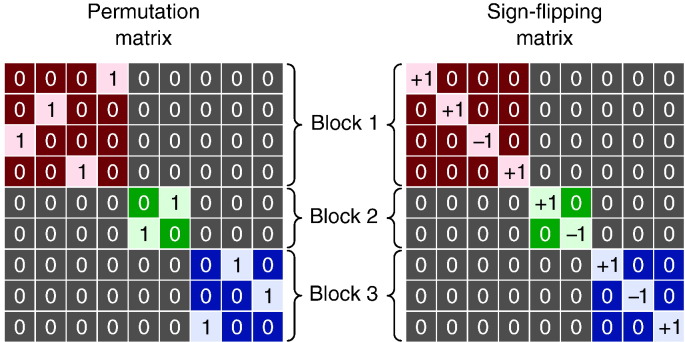
Left: Example of a permutation matrix that shuffles data within block only. The blocks are not required to be of the same size. The elements outside the diagonal blocks are always equal to zero, such that data cannot be swapped across blocks. Right: Example of a sign flipping matrix. Differently than within-block permutation matrices, here sign flipping matrices are transparent to the definitions of the blocks, such that the block definitions do not need to be taken into account, albeit their corresponding variance groups are considered when computing the statistic.

Observations that share the same dependence structure, either assumed or known in advance, can be used to define ebs such that ee are asserted with respect to these blocks only, and the empirical distribution is constructed by permuting exclusively within block, as shown in Fig. 2. Once the blocks have been defined, the regression of nuisance variables and the construction of the reference distribution can follow strategies as Freedman–Lane or Smith, as above. The ise, when applicable, is transparent to this kind of block structure, so that the sign flips occur as under unrestricted exchangeability. For within-block exchangeability, in general each eb corresponds to a vg for the computation of the test statistic. See Appendix C for examples.

##### Whole-block exchangeability

**Fig. 3:**
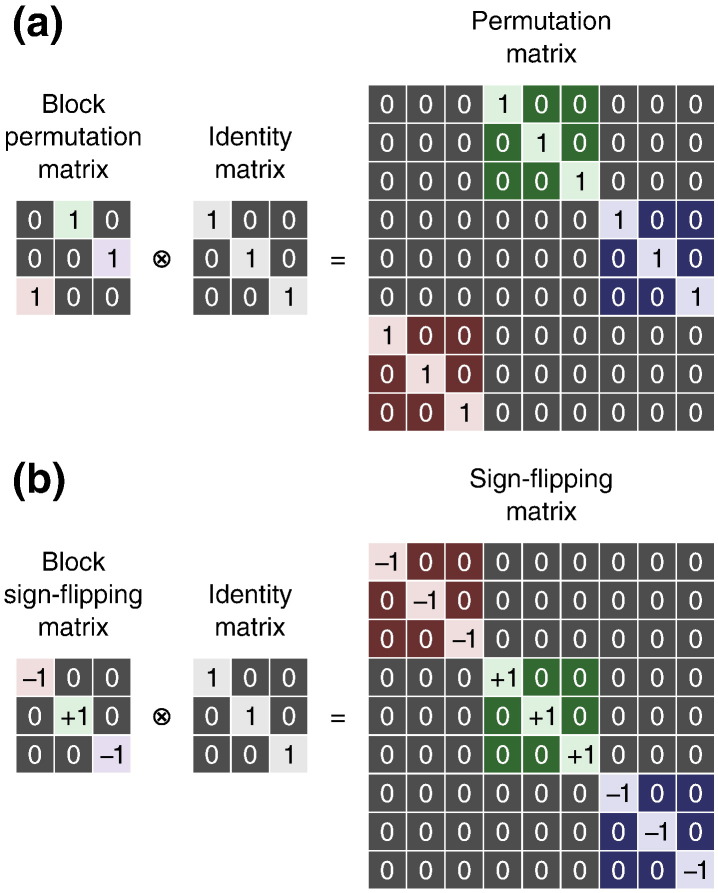
(a) Example of a permutation matrix that shuffles whole blocks of data. The blocks need to be of the same size. (b) Example of a sign flipping matrix that changes the signs of the blocks as a whole. Both matrices can be constructed by the Kronecker product (represented by the symbol ⊗) of a permutation or a sign flipping matrix (with size determined by the number of blocks) and an identity matrix (with size determined by the number of observations per block).

Certain experimental hypotheses may require the comparison of sets of observations to be treated as a whole, being not exchangeable within set. Exchangeability blocks can be constructed such that each include, in a consistent order, all the observations pertaining to a given set and, differently than in within-block exchangeability, here each block is exchanged with the others on their entirety, while maintaining the order of observations within block unaltered. For ise, the signs are flipped for all observations within block at once. Variance groups are not constructed one per block; instead, each vg encompasses one or more observations per block, all in the same order, e.g., one vg with the first observation of each block, another with the second of each block and so on. Consequently, all blocks must be of the same size, and all with their observations ordered consistently, either for ee or for ise. Examples of permutation and sign flipping matrices for whole block permutation are shown in Fig. 3. See Appendix C for examples.

##### Variance groups mismatching exchangeability blocks

While variance groups can be defined implicitly, as above, according to whether within- or whole-block permutation is to be performed, this is not compulsory. In some cases the ebs are defined based on the non-independence between observations, even if the variances across all observations can still be assumed to be identical. See Appendix C for an example using a paired *t*-test.

##### Choice of the configuration of exchangeability blocks

The choice between whole-block and within-block is based on assumptions, or on knowledge about the non-independence between the error terms, as well as on the need to effectively break, at each permutation, the relationship between the data and the regressors of interest. Whole-block can be considered whenever the relationship within subsets of observations, all of the same size, is not identical, but follows a pattern that repeats itself at each subset. Within-block exchangeability can be considered when the relationship between all observations within a subset is identical, even if the subsets are not of the same size, or the relationship itself is not the same for all of them. Whole-block and within-block are straightforward ways to determine the set of valid permutations, but are not the only possibility to determine them, nor are mutually exclusive. Whole-block and within-block can be mixed with each other in various levels of increasing complexity.

##### Choice of the statistic with exchangeability blocks

All the permutation strategies discussed in the previous section can be used with virtually any statistic, the choice resting on particular applications, and constituting a separate topic. The presence of restrictions on exchangeability and variance groups reduces the choices available, though. The statistics *F* and *t*, described in Model and notation, are pivotal and follow known distributions when, among other assumptions, the error terms for all observations are identically distributed. Under these assumptions, all the errors terms can be pooled to compute the residual sum of squares (the term ${\hat{\epsilon }}^{\prime }\hat{\epsilon }$ in Eq. (3)) and so, the variance of the parameter estimates. This forms the basis for parametric inference, and is also useful for non-parametric tests. However, the presence of ebs can be incompatible with the equality of distributions across all observations, with the undesired consequence that pivotality is lost, as shown in the Results. Although these statistics can still be used with permutation methods in general, the lack of pivotality for imaging applications can cause problems for correction of multiple testing. When exchangeability blocks and associated variance groups are present, a suitable statistic can be computed as:

(6) $$G=\frac{{\hat{\psi }}^{\prime }C{\left(C^{\prime }{\left(M^{\prime }\mathrm{WM}\right)}^{-1}C\right)}^{-1}C^{\prime }\hat{\psi }}{\Lambda \cdot \mathrm{rank}\left(C\right)}$$

where **W** is a *N* × *N* diagonal weighting matrix that has elements $W_{\mathrm{nn}}=\frac{\sum_{n^{\prime }\in g_{n}}\;R_{n^{\prime }n^{\prime }}}{{\hat{\epsilon }}_{g_{n}}^{\prime }{\hat{\epsilon }}_{g_{n}}}$, where *g_n_* represents the variance group to which the *n*-th observation belongs, $R_{n^{\prime }n^{\prime }}$ is the *n*′-th diagonal element of the residual forming matrix, and ${\hat{\epsilon }}_{g_{n}}$ is the vector of residuals associated with the same vg.^3^ In other words, each diagonal element of **W** is the reciprocal of the estimated variance for their corresponding group. This variance estimator is equivalent to the one proposed by Horn et al. (1975). The remaining term in Eq. (6) is given by (Welch, 1951):

(7) $$\Lambda =1+\frac{2\left(s-1\right)}{s\left(s+2\right)}\sum_{g}\;\frac{1}{\sum_{n\in g}\;R_{\mathrm{nn}}}{\left(1-\frac{\sum_{n\in g}\;W_{\mathrm{nn}}}{\mathrm{trace}\left(W\right)}\right)}^{2}$$

where s= rank(C) as before. The statistic *G* provides a generalisation of a number of well known statistical tests, some of them summarised in Table 3. When there is only one vg, variance estimates can be pooled across all observations, resulting in Λ = 1 and so, *G* = *F*. If **W** = **V**^− 1^, the inverse of the true covariance matrix, *G* is the statistic for an *F*-test in a weighted least squares model (wls) (Christensen, 2002). If there are multiple variance groups, *G* is equivalent to the *v*^2^ statistic for the problem of testing the means for these groups under no homoscedasticity assumption, i.e., when the variances cannot be assumed to be all equal (Welch, 1951).^4^ If, despite heteroscedasticity, Λ is replaced by 1, *G* is equivalent to the James' statistic for the same problem (James, 1951). When rank(**C**) = 1, and if there are more than one vg, $\mathrm{sign}\left(\hat{\beta }\right)\sqrt{G}$ is the well-known *v* statistic for the Behrens–Fisher problem (Aspin and Welch, 1949; Fisher, 1935b); with only one vg present, the same expression produces the Student's *t* statistic, as shown earlier. If the definition of the blocks and variance groups is respected, all these particular cases produce pivotal statistics, and the generalisation provided by *G* allows straightforward implementation.

### Number of permutations

For a study with *N* observations, the maximum number of possible permutations is *N*!, and the maximum number of possible sign flips is 2*^N^*. However, in the presence of *B* exchangeability blocks that are exchangeable as a whole, the maximum number of possible permutations drops to no more than *B*!, and the maximum number of sign flips to 2*^B^*. For designs where data is only exchangeable within-block, the maximum number of possible permutations is ∏ _*b* = 1_^*B*^ *N_b_*!, where *N_b_* is the number of observations for the *b*-th block, and the maximum number of sign flips continues to be 2*^N^*.

However, the actual number of possible rearrangements may be smaller depending on the null hypothesis, the permutation strategy, or other aspects of the study design. If there are discrete covariates, or if there are ties among continuous regressors, many permutations may not alter the model at all. The maximum number of permutations can be calculated generically from the design matrix observing the number of repeated rows among the regressors of interest for the Freedman–Lane and most other methods, or in **M** for the ter Braak and Manly methods. The maximum number of possible permutations or sign flips, for different restrictions on exchangeability, is shown in Table 4.

Even considering the restrictions dictated by the study design, the number of possible shufflings tends to be very large, even for samples of moderate size, and grows very rapidly as observations are included. When the number of possible rearrangements is large, not all of them need to be performed for the test to be valid (Chung and Fraser, 1958; Dwass, 1957), and the resulting procedure will be approximately exact (Edgington, 1969). The number can be chosen according to the availability of computational resources and considerations about power and precision. The smallest p-value that can be obtained continues to be 1/*J*, where *J* is the number of permutations performed. The precision of permutation p-values may be determined considering the confidence interval around the significance level.

To efficiently avoid permutations that do not change the design matrix, the Algorithm “l” (Knuth, 2005) can be used. This algorithm is simple and has the benefit of generating only permutations that are unique, i.e., in the presence of repeated elements, it correctly avoids synonymous permutations. This is appropriate when enumerating all possible permutations. However, the algorithm produces sequentially permutations that are in lexicographic order. Although this can be advantageous in other settings, here this behaviour can be problematic when running only a subset of P, and has the potential to bias the results. For imaging applications, where there are many points (voxels, vertices, faces) being analysed, it is in general computationally less expensive to shuffle many times a sequence of values and store these permuted sequences, than actually fit the permuted model for all points. As a consequence, the problem with lexicographically ordered permutations can be solved by generating all the possible permutations, and randomly drawing *J* elements from P to do the actual shufflings of the model, or generating random permutations and checking for duplicates. Alternatively, the procedure can be conducted without attention to repeated permutations using simple shuffling of the data. This strategy is known as *conditional Monte Carlo* (cmc) (Pesarin and Salmaso, 2010; Trotter and Tukey, 1956), as each of the random realisations is conditional on the available observed data.

Sign flipping matrices, on the other hand, can be listed using a numeral system with radix 2, and the sign flipped models can be performed without the need to enumerate all possible flips or to appeal to cmc. The simplest strategy is to use the digits 0 and 1 of the binary numeral system, treating 0 as − 1 when assembling the matrix. In a binary system, each sign flipping matrix is also its own numerical identifier, such that avoiding repeated sign flippings is trivial. The binary representation can be converted to and from radix 10 if needed, e.g., to allow easier human readability.

For within-block exchangeability, permutation matrices can be constructed within-block, then concatenated along their diagonal to assemble **P***_j_*, which also has a block structure. The elements outside the blocks are filled with zeros as needed (Fig. 2). The block definitions can be ignored for sign flipping matrices for designs where ise is asserted within-block. For whole-block exchangeability, permutation and sign flipping matrices can be generated by treating each block as an element, and the final **P***_j_* or **S***_j_* are then assembled via Kronecker multiplication by an identity matrix of the same size as the blocks (Fig. 3).

### Multiple testing

Differently than with parametric methods, correction for multiple testing using permutation does not require the introduction of more assumptions. For familywise error rate correction (fwer), the method was described by Holmes et al. (1996). As the statistics *T*_*j*_^∗^ are calculated for each shuffling to build the reference distribution at each point, the maximum value of *T*_*j*_^∗^ across the image, *T*_*j*_^max^, is also recorded for each rearrangement, and its empirical distribution is obtained. For each test in the image, an fwer-corrected p-value can then be obtained by computing the proportion of *T*_*j*_^max^ that is above *T*_0_ for each test. A single fwer threshold can also be applied to the statistical map of *T*_0_ values using the distribution of *T*_*j*_^max^. The same strategy can be used for statistics that combine spatial extent of signals, such as cluster extent or mass (Bullmore et al., 1999), threshold-free cluster enhancement (tfce) (Smith and Nichols, 2009) and others (Marroquin et al., 2011). For these spatial statistics, the effect of lack of pivotality can be mitigated by non-stationarity correction (Hayasaka et al., 2004; Salimi-Khorshidi et al., 2011).

The p-values under the null hypothesis are uniformly distributed in the interval [0,1]. As a consequence, the p-values themselves are pivotal quantities and, in principle, could be used for multiple testing correction as above. The distribution of minimum p-value, *p*_*j*_^min^, instead of *T*_*j*_^max^, can be used. Due to the discreteness of the p-values, this approach, however, entails some computational difficulties that may cause considerable loss of power (Pantazis et al., 2005). Correction based on false-discovery rate (fdr) can be used once the uncorrected p-values have been obtained for each point in the image. Either a single fdr threshold can be applied to the map of uncorrected p-values (Benjamini and Hochberg, 1995; Genovese et al., 2002) or an fdr-adjusted p-value can be calculated at each point (Yekutieli and Benjamini, 1999).

## Evaluation methods

### Choice of the statistic

We conducted extensive simulations to study the behaviour of the common *F* statistic (Eq. 3) as well as of the generalised *G* statistic (Eq. 6), proposed here for use in neuroimaging, in various scenarios of balanced and unbalanced designs and variances for the variance groups. Some of the most representative of these scenarios are shown in Table 5. The main objective of the simulations was to assess whether these statistics would retain their distributions when the variances are not equal for each sample. Within each scenario, 3 or 5 different configurations of simulated variances were tested, pairwise, for the equality of distributions using the two-sample Kolmogorov–Smirnov test (ks) (Press et al., 1992), with a significance level *α* = 0.05, corrected for multiple testing within each scenario using the Bonferroni correction, as these tests are independent.

For each variance configuration, 1000 voxels containing normally distributed random noise, with zero expected mean, were simulated and tested for the null hypothesis of no difference between the means of the groups. The empirical distribution of the statistic for each configuration was obtained by pooling the results for the simulated voxels, then compared with the ks test. The process was repeated 1000 times, and the number of times in which the distributions were found to be significantly different from the others in the same scenario was recorded. Confidence intervals (95%) were computed using the Wilson method (Wilson, 1927).

By comparing the distributions of the same statistic obtained in different variance settings, this evaluation strategy mimics what is observed when the variances for each voxel varies across space in the same imaging experiment, e.g., *(a)*, *(b)* and *(c)* in Table 5 could be different voxels in the same image. The statistic must be robust to these differences and retain its distributional properties, even if assessed non-parametrically, otherwise fwer using the distribution of the maximum statistic is compromised. The same applies to multiple testing that combines more than one imaging modality.

In addition, the same scenarios and variance configurations were used to assess the proportion of error type i and the power of the *F* and *G* statistics. To assess power, a simulated signal was added to each of the groups; for the scenarios with two groups, the true *ψ* was defined as [0 -1]′, whereas for the scenarios with four groups, it was defined as [0 -0.33 -0.67 -1]′. In either case, the null hypothesis was that the group means were all equal. Significance values were computed using 1000 permutations, with *α* = 0.05, and 95% confidence intervals were calculated using the Wilson method.

### Permutation strategies

We compared the 10 methods described in Table 2 simulating different regression scenarios. The design considered one regressor of interest, **x**_1_, and two regressors of no interest, **z**_1_ and **z**_2_, **z**_2_ being a column-vector of just ones (intercept). The simulation scenarios considered different sample sizes, *N* = {12, 24, 48, 96}; different combinations for continuous and categorical **x**_1_ and **z**_1_; different degrees of correlation between **x**_1_ and **z**_1_, *ρ* = {0, 0.8}; different sizes for the regressor of interest, *β*_1_ = {0, 0.5}; and different distributions for the error terms, ϵ, as normal (*μ* = 0, *σ*^2^ = 1), uniform $\left(\left[-\sqrt{3},+\sqrt{3}\right]\right)$, exponential (*λ* = 1) and Weibull (*λ* = 1, *k* = 1/3). The coefficients for the first regressor of no interest and for the intercept were kept constant as *γ*_1_ = 0.5 and *γ*_2_ = 1 respectively, and the distributions of the errors were shifted or scaled as needed to have expected zero mean and expected unit variance.

The continuous regressors were constructed as a linear trend ranging from − 1 to + 1 for **x**_1_, and the square of this trend, mean-centred, for **z**_1_. For this symmetric range around zero for **x**_1_, this procedure causes **x**_1_ and z_1_ to be orthogonal and uncorrelated. For the discrete regressors, a vector of *N*/2 ones and *N*/2 negative ones was used, the first *N*/2 values being only + 1 and the remaining − 1 for **x**_1_, whereas for **z**_1_, the first and last *N*/4 were − 1 and the *N*/2 middle values were + 1. This procedure also causes **x**_1_ and **z**_1_ to be orthogonal and uncorrelated. For each different configuration, 1000 simulated vectors **Y** were constructed as **Y** = [**x**_1_ **z**_1_ **z**_2_][*β*_1_ *γ*_1_ *γ*_2_]′ + ϵ.

Correlation was introduced in the regression models through Cholesky decomposition of the desired correlation matrix **K**, such that **K** = **L**′**L**, then defining the regressors by multiplication by **L**, i.e., [**x**_1_^*ρ*^ **z**_1_^*ρ*^] = [**x**_1_ **z**_1_]**L**. The unpartitioned design matrix was constructed as **M** = [**x**_1_^*ρ*^**z**_1_^*ρ*^**z**_2_]. A contrast **C** = [1 0 0]′ was defined to test the null hypothesis H=_0_ : **C**′*ψ* = *β*_1_ = 0. This contrast tests only the first column of the design matrix, so partitioning **M** = [**X Z**] using the scheme shown in Appendix A might seem unnecessary. However, we wanted to test also the effect of non-orthogonality between columns of the design matrix for the different permutation methods, with and without the more involved partitioning scheme shown in the Appendix. Using a single variance configuration across all observations in each simulation, modelling a single variance group, and with rank(C) = 1, the statistic used was the Student's *t* (Table 3), a particular case of the *G* statistic. Permutation, sign flipping, and permutation with sign flipping were tested. Up to 1000 permutations and/or sign flippings were performed using cmc, being less when the maximum possible number of shufflings was not large enough. In these cases, all the permutations and/or sign flippings were performed exhaustively.

Error type i was computed using *α* = 0.05 for configurations that used *β*_1_ = 0. The other configurations were used to examine power. As previously, confidence intervals (95%) were estimated using the Wilson method.

## Results

### Choice of the statistic

**Fig. 4:**
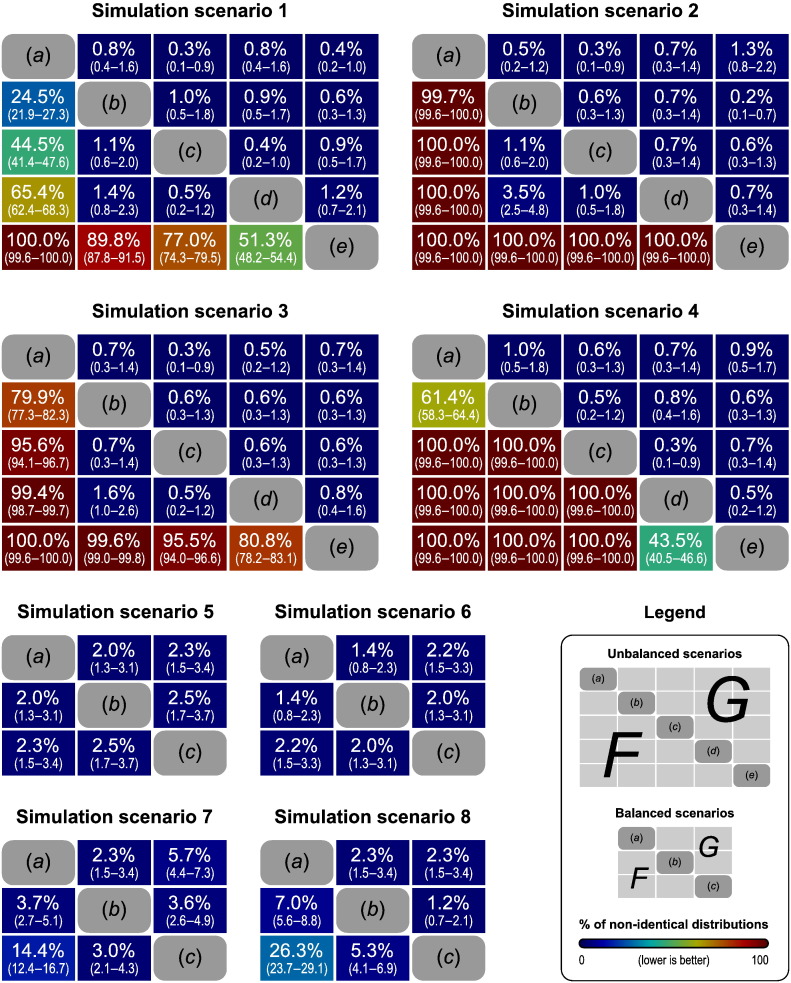
Heatmaps for the comparison of the distributions obtained under different variance settings for identical sample sizes. In each map, the cells below the main diagonal contain the results for the pairwise *F* statistic, and above, for the *G* statistic. The percentages refer to the fraction of the 1000 tests in which the distribution of the statistic for one variance setting was found different than for another in the same simulation scenario. Each variance setting is indicated by letters (*a*–*e*), corresponding to the same letters in Table 5. Smaller percentages indicate robustness of the statistic to heteroscedasticity. Confidence intervals (95%) are shown in parenthesis.

Fig. 4 shows heatmaps with the results of the pairwise comparisons between variance configurations, within each of the simulation scenarios presented in Table 5, using either *F* or *G* statistic. For unbalanced scenarios with only two samples (simulation scenarios 1 to 3), and with modest variance differences between groups (configurations *b* to *d*), the *F* statistic often retained its distributional properties, albeit less often than the *G* statistic. For large variance differences, however, this relative stability was lost for *F*, but not for *G* (*a* and *e*). Moreover, the inclusion of more groups (scenario 4), with unequal sample sizes, caused the distribution of the *F* statistic to be much more sensitive to heteroscedasticity, such that almost always the ks test identified different distributions across different variance configurations. The *G* statistic, on the other hand, remained robust to heteroscedasticity even in these cases. As one of our reviewers highlighted, a variance ratio of 15:1 (as used in Scenarios 4, 7 and 8) may seem somewhat extreme, but given the many thousands, often millions, of voxels in an image, it is not unreasonable to suspect that such large variance differences may exist across at least some of them.

In balanced designs, either with two (simulation scenarios 5 and 6) or more (scenarios 7 and 8) groups, the *F* statistic had a better behaviour than in unbalanced cases. For two samples of the same size, there is no difference between *F* and *G*: both have identical values and produce the same permutation p-values.^5^ For more than two groups, the *G* statistic behaved consistently better than *F*, particularly for large variance differences.

These results suggest that the *G* statistic is more appropriate under heteroscedasticity, with balanced or unbalanced designs, as it preserves its distributional properties, indicating more adequacy for use with neuroimaging. The *F* statistic, on the other hand, does not preserve pivotality but can, nonetheless, be used under heteroscedasticity when the groups have the same size.

With respect to error type i, both *F* and *G* resulted in similar amount of false positives when assessed non-parametrically. The *G* yielded generally higher power than *F*, particularly in the presence of heteroscedasticity and with unequal sample sizes. These results are presented in Table 6.

### Permutation strategies

The different simulation parameters allowed 1536 different regression scenarios, being 768 without signal and 768 with signal; a summary is shown in Table 7, and some of the most representative in Table 8. In “well behaved” scenarios, i.e., large number of observations, orthogonal regressors and normally distributed errors, all methods tended to behave generally well, with adequate control over type i error and fairly similar power. However, performance differences between the permutation strategies shown in Table 2 became more noticeable as the sample sizes were decreased and skewed errors were introduced.

Some of the methods are identical to each other in certain circumstances. If **X** and **Z** are orthogonal, Draper–Stoneman and Smith are equivalent. Likewise under orthogonality, Still–White produces identical regression coefficients as Freedman–Lane, although the statistic will only be the same if the loss in degrees of freedom due to **Z** is taken into account, something not always possible when the data has already been residualised and no information about the original nuisance variables is available. Nonetheless, the two methods remain asymptotically equivalent as the number of observations diverges from the number of nuisance regressors.

#### Sample size

Increasing the sample size had the effect of approaching the error rate closer to the nominal level *α* = 0.05 for all methods in virtually all parameter configurations. For small samples, most methods were slightly conservative, whereas Still–White and Kennedy were anticonservative and often invalid, particularly if the distributions of the errors were skewed.

#### Continuous or categorical regressors of interest

For all methods, using continuous or categorical regressors of interest did not produce remarkable differences in the observed proportions of type i error, except if the distribution of the errors was skewed and sign flipping was used (in violation of assumptions), in which case Manly and Huh–Jhun methods showed erratic control over the amount of errors.

#### Continuous or categorical nuisance regressors

The presence of continuous or categorical nuisance variables did not substantially interfere with either control over error type i or power, for any of the methods, except in the presence of correlated regressors.

#### Degree of non-orthogonality and partitioning

All methods provided relatively adequate control over error type i in the presence of a correlated nuisance regressor, except Still–White (conservative) and Kennedy (inflated rates). The partitioning scheme mitigated the conservativeness of the former, and the anticonservativeness of the latter.

#### Distribution of the errors

Different distributions did not substantially improve or worsen error rates when using permutation alone. Still–White and Kennedy tended to fail to control error type i in virtually all situations. Sign flipping alone, when used with asymmetric distributions (in violation of assumptions), required larger samples to allow approximately exact control over the amount of error type i. In these cases, and with small samples, the methods Draper–Stoneman, Manly and Huh–Jhun tended to display erratic behaviour, with extremes of conservativeness and anticonservativeness depending on the other simulation parameters. The same happened with the parametric method. Freedman–Lane and Smith methods, on the other hand, tended to have a relatively constant and somewhat conservative behaviour in these situations. Permutation combined with sign flipping generally alleviated these issues where they were observed.

From all the methods, the Freedman–Lane and Smith were those that performed better in most cases, and with their 95% confidence interval covering the desired error level of 0.05 more often than any of the other methods. The Still–White and Kennedy methods did not generally control the error type i for most of the simulation parameters, particularly for smaller sample sizes. On the other hand, with a few exceptions, the Freedman–Lane and the Smith methods effectively controlled the error rates in most cases, even with skewed errors and sign flipping, being, at worst, conservative or only slightly above the nominal level. All methods were, overall, similarly powerful, with only marginal differences among those that were on average valid.

## Discussion

Criteria to accept or reject a hypothesis should, ideally, be powerful to detect true effects, and insensitive to nuisance factors (Box and Andersen, 1955). A compromise between these features is often present and, in neuroimaging applications, this compromise gains new contours. First, different imaging modalities do not follow necessarily the same set of assumptions regarding distributions under the null or the covariance between tests across the brain, with the consequence that both false positives and false negatives can arise when parametric tests are used haphazardly. Second, in neuroimaging it is necessary to address the multiple testing problem. Parametric methods require an even larger set of assumptions to deal with this problem, amplifying the risk of errors when these supernumerary assumptions are not met. Third, under non-random sampling, as is common in case–control studies, the very presence of the features under investigation may compromise the assumptions on which parametric tests depend. For all these reasons, parametric methods are more likely to fail as candidates to provide a general statistical framework for the current variety of imaging modalities for research applications, where not only the assumptions may not be met, but also where robustness may be seen as a key factor. Permutation methods are a viable alternative, flexible enough to accommodate several experimental needs. Further to all this, our simulations showed similar and sometimes higher power compared to the parametric approach.

### Permutation tests

Permutation tests require very few assumptions about the data and, therefore, can be applied in a wider variety of situations than parametric tests. Moreover, only a few of the most common parametric assumptions need to hold for non-parametric tests to be valid. The assumptions that are eschewed include, for instance, the need of normality for the error terms, the need of homoscedasticity and the need of random sampling. With a very basic knowledge of sample properties or of the study design, errors can be treated as exchangeable (ee) and/or independent and symmetric (ise) and inferences that otherwise would not be possible with parametric methods become feasible. Furthermore, permutation tests permit the use of the very same regression and hypothesis testing framework, even with disparate imaging modalities, without the need to verify the validity of parametric assumptions for each of them. The ise can be an alternative to ee when the errors themselves can be considered exchangeable, but the design is not affected by permutations, as for one-sample tests. And if the assumptions for ee and ise are both met, permutation and sign flipping can both be performed to construct the empirical distribution.

The justification for permutation tests has, moreover, more solid foundations than their parametric counterparts. While the validity of parametric tests relies on random sampling, permutation tests have their justification on the idea of random allocation of experimental units, with no reference to any underlying population (Edgington, 1995; Manly, 2007). This aspect has a key importance in biomedical research — including neuroimaging — where only a small minority of studies effectively use random population sampling. Most experimental studies need to use the subjects that are available in a given area, and who accept to participate (e.g. patients of a hospital or students of a university near where the mri equipment is installed). True random sampling is rarely achieved in real applications because, often and for different reasons, selection criteria are not truly unbiased (Ludbrook and Dudley, 1998; Pesarin and Salmaso, 2010). Non-parametric methods allow valid inferences to be performed in these scenarios.

### Pivotal statistics

In addition, permutation methods have the remarkable feature of allowing the use of non-standard statistics, or for which closed mathematical forms have not been derived, even asymptotically. Statistics that can be used include, for instance, those based on ranks of observations (Brunner and Munzel, 2000; Rorden et al., 2007), derived from regression methods other than least squares (Cade and Richards, 1996) or that are robust to outliers (Sen, 1968; Theil, 1950). For imaging applications, statistics that can be considered include the pseudo-*t* statistic after variance smoothing (Holmes et al., 1996), the mass of connected voxels (Bullmore et al., 1999), threshold-free cluster enhancement (tfce) (Smith and Nichols, 2009), as well as cases in which the distribution of the statistic may lie in a gradient between distributions, each of them with known analytical forms (Winkler et al., 2012). The only requirement, in the context of neuroimaging, is that these statistics retain their distributional properties irrespective to unknown parameters.

Indeed, a large part of the voluminous literature on statistical tests when the errors cannot be assumed to be homoscedastic is concerned with the identification of the asymptotic distribution of the statistics, its analytical form, and the consequences of experimental scenarios that include unbalancedness and/or small samples. This is true even considering that in parametric settings, the statistics are invariably chosen such that their sampling distribution is independent of underlying and unknown population parameters. Permutation tests render all these issues irrelevant, as the asymptotic properties of the distributions do not need to be ascertained. For imaging, all that is needed is that the distribution remains invariant to unknown population parameters, i.e., the statistic needs to be pivotal. Parameters of the distribution proper do not need to be known, nor the distribution needs to be characterised analytically. The proposed statistic *G*, being a generalisation over various tests that have their niche applications in parametric settings, is appropriate for use with the general linear model and with a permutation framework, for being pivotal and easily implementable using simple matrix operations. Moreover, as the simulations showed, this statistic is not less powerful than the commonly used *F* statistic.

### Permutation strategies

From the different permutation strategies presented in Table 2, the Freedman–Lane and the Smith methods provided the most adequate control of type i error across the various simulation scenarios. This is in line with the study by Anderson and Legendre (1999), who found that the Freedman–Lane method is the most accurate and powerful in various different models. The Smith method was a somewhat positive surprise, not only for the overall very good performance in our simulations, but also because this method has not been extensively evaluated in previous literature, is computationally simple, and has an intuitive appeal.

Welch (1990) commented that the Freedman–Lane procedure would violate the ancillarity principle, as the permutation procedure would destroy the relationship between **X** and **Z**, even if these are orthogonal. Notwithstanding, even with ancillarity violated, this and other methods perform satisfactorily well as shown by the simulations.

Freedman and Lane (1983) described their method as having a “non-stochastic” interpretation, and so, that the computed p-value would be a descriptive statistic. On the contrary, we share the same view expressed by Anderson and Legendre (1999), that the rationale for the test and the procedure effectively produces a p-value that can be interpreted as a true probability for the underlying model.

Regarding differences between the methods, and even though for this study we did not evaluate the effect of extremely strong signals or of outliers, it is worth commenting that previous research have shown that the Freedman–Lane method is relatively robust to the presence of extreme outliers, whereas the ter Braak tends to become more conservative in these cases (Anderson and Legendre, 1999). The ter Braak method, however, was shown to be more robust to extremely strong signals in the data, situations in which signal may “leak” into the permutation distribution with the Freedman–Lane method (Salimi-Khorshidi et al., 2011).

It should be noted that the Still–White method, as implemented for these simulations, used the model containing only the regressors of interest when computing the statistic as shown in Table 2. It is done in this way to emulate what probably is its more common use, i.e., rearrange the data that has already been residualised from nuisance, and when the nuisance regressors are no longer available. Had the full model been used when computing the statistic, it is possible that this method might have performed somewhat similarly as Freedman–Lane, specially for larger samples. Moreover, neither the original publication (Still and White, 1981), nor a related method published shortly after (Levin and Robbins, 1983), specify how the degrees of freedom should be treated when computing the statistic in a generic formulation as we present here.

With respect to non-independent measurements, these are addressed by means of treating the observations as weakly exchangeable (Good, 2002), that is, allowing only the permutations that respect the covariance structure of the data and maintain its joint distribution intact. Not all null hypotheses can be addressed in this way, however, as the restricted set of permutations may not sufficiently disrupt the relationship between the regressors of interest and the observed data without appealing to sign flipping, and even so, only if the ise assumptions are met. The use of a restricted set of permutations, that is, a subset of all otherwise possible permutations, allows various studies involving non-independent measurements to be adequately analysed (Good, 2005; Manly, 2007). However, it should be emphasised that not all designs that include repeated measurements can be trivially analysed, and if the study is not adequately planned, it may become impossible to draw conclusions using permutation methods — albeit the same may likely apply to parametric tests. We note that using permutations that respect the data structure, without the need to explicitly model it, is a great benefit of the methods as proposed.

Finally, although non-parametric methods are generally considered less powerful than their parametric counterparts, we found in the simulations performed that most of the permutation methods are not substantially less powerful than the parametric method, and sometimes are even more powerful, even when the assumptions of the latter are met. With the availability of computing power and reliable software implementation, there is almost no reason for not using these permutation methods.

## Conclusion

We presented a generic framework that allows permutation inference using the general linear model with complex experimental designs, and which depends only on the weak requirements of exchangeable or independent and symmetric errors, which define permutations, sign flippings, or both. Structured dependence between observations is addressed through the definition of exchangeability blocks. We also proposed a statistic that is robust to heteroscedasticity, can be used for multiple-testing correction, and can be implemented easily with matrix operations. Based on evaluations, we recommend the Freedman–Lane and the Smith methods to construct the empirical distribution, and use Freedman–Lane in the randomise algorithm (Appendix B).

## Figures and Tables

**Figure:**
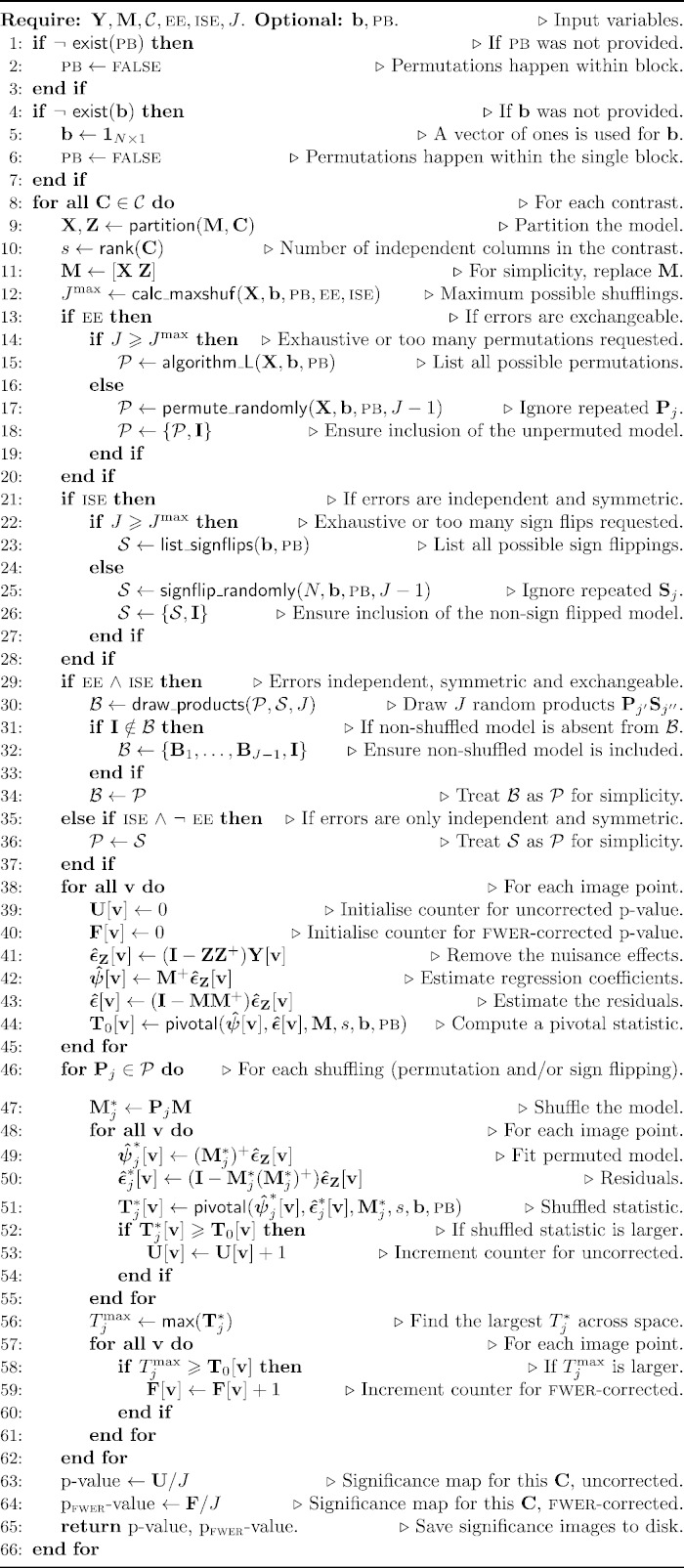


**Table 9:** Coding of the design matrix, exchangeability blocks and variance groups for Example 1. Under unrestricted exchangeability, all subjects are assigned to a single block, and with identical variances, all to a single variance group. The regressor **m_1_** codes for the overall mean, whereas **m_2_** codes for handedness.

Coded data (**Y**)	eb	vg	Model (**M**)	
			**m_1_**	**m_2_**
Subject 1	1	1	1	*h*_1_
Subject 2	1	1	1	*h*_2_
Subject 3	1	1	1	*h*_3_
Subject 4	1	1	1	*h*_4_
Subject 5	1	1	1	*h*_5_
Subject 6	1	1	1	*h*_6_
Subject 7	1	1	1	*h*_7_
Subject 8	1	1	1	*h*_8_
Subject 9	1	1	1	*h*_9_
Subject 10	1	1	1	*h*_10_
Subject 11	1	1	1	*h*_11_
Subject 12	1	1	1	*h*_12_
Contrast 1 (**C**_1_′)			+ 1	0
Contrast 2 (**C**_2_′)			− 1	0

**Table 10:** Coding for Example 2. Under unrestricted exchangeability, all subjects are assigned to a single block. The regressors **m_1_** and **m_2_** code for the experimental groups, **m_3_** and **m_4_** for age and sex.

Coded data (**Y**)	eb	vg	Model (**M**)			
			**m_1_**	**m_2_**	**m_3_**	**m_4_**
Subject 1	1	1	1	0	*a*_1_	*s*_1_
Subject 2	1	1	1	0	*a*_2_	*s*_2_
Subject 3	1	1	1	0	*a*_3_	*s*_3_
Subject 4	1	1	1	0	*a*_4_	*s*_4_
Subject 5	1	1	1	0	*a*_5_	*s*_5_
Subject 6	1	1	1	0	*a*_6_	*s*_6_
Subject 7	1	1	0	1	*a*_7_	*s*_7_
Subject 8	1	1	0	1	*a*_8_	*s*_8_
Subject 9	1	1	0	1	*a*_9_	*s*_9_
Subject 10	1	1	0	1	*a*_10_	*s*_10_
Subject 11	1	1	0	1	*a*_11_	*s*_11_
Subject 12	1	1	0	1	*a*_12_	*s*_12_
Contrast 1 (**C**_1_′)			+ 1	− 1	0	0
Contrast 2 (**C**_2_′)			− 1	+ 1	0	0

**Table 11:** Coding of the design matrix exchangeability blocks and variance groups for Example 3. Observations are exchangeable only within subject, and variance can be estimated considering all observations as a single group. The regressor **m_1_** codes for treatment, whereas **m_2_** to **m_7_** code for subject-specific mean.

Coded data (**Y**)	eb	vg	Model (**M**)						
			**m_1_**	**m_2_**	**m_3_**	**m_4_**	**m_5_**	**m_6_**	**m_7_**
Subj. 1, obs. 1	1	1	+ 1	1	0	0	0	0	0
Subj. 2, obs. 1	2	1	+ 1	0	1	0	0	0	0
Subj. 3, obs. 1	3	1	+ 1	0	0	1	0	0	0
Subj. 4, obs. 1	4	1	+ 1	0	0	0	1	0	0
Subj. 5, obs. 1	5	1	+ 1	0	0	0	0	1	0
Subj. 6, obs. 1	6	1	+ 1	0	0	0	0	0	1
Subj. 1, obs. 2	1	1	− 1	1	0	0	0	0	0
Subj. 2, obs. 2	2	1	− 1	0	1	0	0	0	0
Subj. 3, obs. 2	3	1	− 1	0	0	1	0	0	0
Subj. 4, obs. 2	4	1	− 1	0	0	0	1	0	0
Subj. 5, obs. 2	5	1	− 1	0	0	0	0	1	0
Subj. 6, obs. 2	6	1	− 1	0	0	0	0	0	1
Contrast 1 (**C**_1_′)			+ 1	0	0	0	0	0	0
Contrast 2 (**C**_2_′)			− 1	0	0	0	0	0	0

**Table 12:** Coding of the design matrix and exchangeability blocks for Example 4. As the group variances cannot be assumed to be the same, each group constitutes an EB and VG; sign flippings happen within block. The regressors **m_1_** and **m_2_** code for the experimental groups, **m_3_** and **m_4_** for age and sex.

Coded data (**Y**)	eb	vg	Model (**M)**			
			**m_1_**	**m_2_**	**m_3_**	**m_4_**
Subject 1	1	1	1	0	*a*_1_	*s*_1_
Subject 2	1	1	1	0	*a*_2_	*s*_2_
Subject 3	1	1	1	0	*a*_3_	*s*_3_
Subject 4	1	1	1	0	*a*_4_	*s*_4_
Subject 5	1	1	1	0	*a*_5_	*s*_5_
Subject 6	1	1	1	0	*a*_6_	*s*_6_
Subject 7	2	2	0	1	*a*_7_	*s*_7_
Subject 8	2	2	0	1	*a*_8_	*s*_8_
Subject 9	2	2	0	1	*a*_9_	*s*_9_
Subject 10	2	2	0	1	*a*_10_	*s*_10_
Subject 11	2	2	0	1	*a*_11_	*s*_11_
Subject 12	2	2	0	1	*a*_12_	*s*_12_
Contrast 1 (**C**_1_′)			+ 1	− 1	0	0
Contrast 2 (**C**_2_′)			− 1	+ 1	0	0

**Table 13:** Coding for Example 5. The different variances restrict exchangeability for within same sex only, and two exchangeability blocks are defined, for shuffling within block. The regressors **m_1_** and **m_2_** code for group (patients and controls), whereas **m_3_** codes for sex.

Coded data (**Y**)	eb	vg	Model (**M**)		
			**m_1_**	**m_2_**	**m_3_**
Subject 1	1	1	1	0	1
Subject 2	1	1	1	0	1
Subject 3	1	1	1	0	1
Subject 4	2	2	1	0	− 1
Subject 5	2	2	1	0	− 1
Subject 6	2	2	1	0	− 1
Subject 7	1	1	0	1	1
Subject 8	1	1	0	1	1
Subject 9	1	1	0	1	1
Subject 10	2	2	0	1	− 1
Subject 11	2	2	0	1	− 1
Subject 12	2	2	0	1	− 1
Contrast 1 (**C**_1_′)			1	− 1	0
Contrast 2 (**C**_2_′)			− 1	1	0

**Table 14:** Coding of the design matrix, exchangeability blocks and variance groups for Example 6. Shufflings happen for the blocks as a whole, and variances are not assumed to be the same across all timepoints.

Coded data (Y)	eb	vg	Model (**M**)					
			**m_1_**	**m_2_**	**m_3_**	**m_4_**	**m_5_**	**m_6_**
Subject 1, Timepoint 1	1	1	*a*_11_	0	1	0	0	0
Subject 1, Timepoint 2	1	2	*a*_12_	0	1	0	0	0
Subject 1, Timepoint 3	1	3	*a*_13_	0	1	0	0	0
Subject 2, Timepoint 1	2	1	*a*_21_	0	0	1	0	0
Subject 2, Timepoint 2	2	2	*a*_22_	0	0	1	0	0
Subject 2, Timepoint 3	2	3	*a*_23_	0	0	1	0	0
Subject 3, Timepoint 1	3	1	0	*a*_31_	0	0	1	0
Subject 3, Timepoint 2	3	2	0	*a*_32_	0	0	1	0
Subject 3, Timepoint 3	3	3	0	*a*_33_	0	0	1	0
Subject 4, Timepoint 1	4	1	0	*a*_41_	0	0	0	1
Subject 4, Timepoint 2	4	2	0	*a*_42_	0	0	0	1
Subject 4, Timepoint 3	4	3	0	*a*_43_	0	0	0	1
Contrast 1 (**C**_1_′)			1	− 1	0	0	0	0
Contrast 2 (**C**_2_′)			− 1	1	0	0	0	0

**Table 1:** Compared with parametric methods, permutation tests relax a number of assumptions and can be used in a wider variety of situations. Some of these assumptions can be further relaxed with the definition of exchangeability blocks.

Assumptions	ee	ise	Parametric
*With respect to the dependence structure between error terms:*			
Independent	✓	✓	✓
Non-independent, exchangeable	✓	✗	✗
Non-independent, non-exchangeable	✗	✗	✗

*With respect to the distributions of the error terms:*			
Normal, identical	✓	✓	✓
Symmetrical, identical	✓	✓	✗
Symmetrical, non-identical	✗	✓	✗
Skewed, identical	✓	✗	✗
Skewed, non-identical	✗	✗	✗

✓Can be used directly if the assumptions regarding dependence structure and distribution of the error terms are both met. ✗Cannot be used directly, or can be used in particular cases.

**Table 2:** A number of methods are available to obtain parameter estimates and construct a reference distribution in the presence of nuisance variables.

Method	Model
Draper–Stoneman^a^	**Y** = **PX***β* + **Z***γ* + ϵ
Still–White^b^	**PR**_**Z**_**Y** = **X***β* + ϵ
Freedman–Lane^c^	(**PR**_**Z**_ + **H**_**Z**_)**Y** = **X***β* + **Z***γ* + ϵ
Manly^d^	**PY** = **X***β* + **Z***γ* + ϵ
ter Braak^e^	(**PR**_**M**_ + **H**_**M**_)**Y** = **X***β* + **Z***γ* + ϵ
Kennedy^f^	**PR**_**Z**_**Y** = **R**_**Z**_**X***β* + ϵ
Huh–Jhun^g^	**PQ**′**R**_**Z**_**Y** = **Q**′**R**_**Z**_**X***β* + ϵ
Smith^h^	**Y** = **PR**_**Z**_**X***β* + **Z***γ* + ϵ
Parametric^i^	**Y** = **Xβ** + **Zγ** +ϵ, ϵ ∼ **N**(**0**, **σ**^**2**^**I**)

a Draper and Stoneman (1966). This method was called “Shuffle Z” by (Kennedy, 1995), and using the same notation adopted here, it would be called “Shuffle X”.

b Gail et al. (1988); Levin and Robbins (1983); Still and White (1981). Still and White considered the special anova case in which **Z** are the main effects and **X** the interaction.

c Freedman and Lane (1983).

d Manly (1986); Manly (2007).

e ter Braak (1992). The null distribution for this method considers ${\hat{\beta }}_{j}^{*}=\hat{\beta }$, i.e., the permutation happens under the alternative hypothesis, rather than the null.

f Kennedy (1995); Kennedy and Cade (1996). This method was referred to as “Residualize both Y and Z” in the original publication, and using the same notation adopted here, it would be called “Residualize both Y and X”.

g Huh and Jhun (2001); Jung et al. (2006); Kherad-Pajouh and Renaud (2010). **Q** is a *N′* × *N*′ matrix, where *N*′ is the rank of **R_Z_**. **Q** is computed through Schur decomposition of **R_Z_**, such that *R*_**Z**_ = **QQ**′ and $I_{N^{\prime }\times N^{\prime }}=Q^{\prime }Q$. For this method, **P** is *N*′ × *N*′. From the methods in the table, this is the only that cannot be used directly under restricted exchangeability, as the block structure is not preserved.

h The Smith method consists of orthogonalization of **X** with respect to **Z**. In the permutation and multiple regression literature, this method was suggested by a referee of O'Gorman (2005), and later presented by Nichols et al. (2008) and discussed by Ridgway (2009).

i The parametric method does not use permutations, being instead based on distributional assumptions. For all the methods, the left side of the equations contains the data (regressand), the right side the regressors and error terms. The unpermuted models can be obtained by replacing **P** for **I**. Even for the unpermuted models, and even if **X** and **Z** are orthogonal, not all these methods produce the same error terms ϵ. This is the case, for instance, of the Kennedy and Huh–Jhun methods. Under orthogonality between **X** and **Z**, some regression methods are equivalent to each other.

**Table 3:** The statistic *G* provides a generalisation for a number of well known statistical tests.

	rank(**C**) = 1	rank(**C**) > 1
Homoscedastic errors, unrestricted exchangeability	Square of Student's *t*	*F*-ratio
Homoscedastic within vg, restricted exchangeability	Square of Aspin–Welch *v*	Welch's *v*^2^

**Table 4:** Maximum number of unique permutations considering exchangeability blocks.

Exchangeability	ee	ise
Unrestricted	*N*!	2*^N^*
Unrestricted, repeated rows	$N!\prod_{m=1}^{M}\;\frac{1}{N_{m}!}$	2*^N^*
Within-block	$\prod_{b=1}^{B}\;N_{b}!$	2*^N^*
Within-block, repeated rows	$\prod_{b=1}^{B}\;N_{b}!\prod_{m=1}^{M|b}\;\frac{1}{N_{m|b}!}$	2*^N^*
Whole-block	*B*!	2*^B^*
Whole-block, repeated blocks	$B!\prod_{\tilde{m}=1}^{\tilde{M}}\;\frac{1}{N_{\tilde{m}}!}$	2*^B^*

*B* Number of exchangeability blocks (eb). *M* Number of distinct rows in **X**. *M*|*b* Number of distinct rows in **X** within the *b*-th block. $\tilde{M}$ Number of distinct blocks of rows in **X**. *N* Number of observations. *N_b_* Number of observations in the *b*-th block. *N_m_* Number of times each of the *M* distinct rows occurs in **X**. *N*_*m*|*b*_ Number of times each of the *m*-th unique row occurs within the *b*-th block. $N_{\tilde{m}}$ Number of times each of the $\tilde{M}$ distinct blocks occurs in **X**.

**Table 5:** The eight different simulation scenarios, each with its own same sample sizes and different variances. The distributions of the statistic (*F* or *G*) for each pair of variance configuration within scenario were compared using the KS test. The letters in the last column (marked with a star, ⋆) indicate the variance configurations represented in the pairwise comparisons shown in Fig. 4 and results shown in Table 6.

Simulation scenario	Sample sizes for each vg	Variances for each vg	⋆
1	8, 4	5, 1	(*a*)
		1.2, 1	(*b*)
		1, 1	(*c*)
		1, 1.2	(*d*)
		1, 5	(*e*)
2	20, 5	5, 1	(*a*)
		1.2, 1	(*b*)
		1, 1	(*c*)
		1, 1.2	(*d*)
		1, 5	(*e*)
3	80, 30	5, 1	(*a*)
		1.2, 1	(*b*)
		1, 1	(*c*)
		1, 1.2	(*d*)
		1, 5	(*e*)
4	40, 30, 20, 10	15, 10, 5, 1	(*a*)
		3.6, 2.4, 1.2, 1	(*b*)
		1, 1, 1, 1	(*c*)
		1, 1.2, 2.4, 3.6	(*d*)
		1, 5, 10, 15	(*e*)
5	4, 4	1, 1	(*a*)
		1, 1.2	(*b*)
		1, 5	(*c*)
6	20, 20	1, 1	(*a*)
		1, 1.2	(*b*)
		1, 5	(*c*)
7	4, 4, 4, 4	1, 1, 1, 1	(*a*)
		1, 1.2, 2.4, 3.6	(*b*)
		1, 5, 10, 15	(*c*)
8	20, 20, 20, 20	1, 1, 1, 1	(*a*)
		1, 1.2, 2.4, 3.6	(*b*)
		1, 5, 10, 15	(*c*)

**Table 6:** Proportion of error type I and power (%) for the statistics *F* and *G* in the various simulation scenarios and variance configurations shown in Table 5. Confidence intervals (95%) are shown in parenthesis.

Simulation scenario	⋆	Proportion of error type i		Power	
		*F*	*G*	*F*	*G*
1	(*a*)	5.9 (4.6–7.5)	6.1 (4.8–7.8)	20.1 (17.7–22.7)	23.8 (21.3–26.5)
	(*b*)	4.9 (3.7–6.4)	5.3 (4.1–6.9)	28.3 (25.6–31.2)	31.9 (29.1–34.9)
	(*c*)	4.7 (3.6–6.2)	4.5 (3.4–6.0)	29.3 (26.6–32.2)	32.6 (29.8–35.6)
	(*d*)	4.9 (3.7–6.4)	4.6 (3.5–6.1)	29.9 (27.1–32.8)	32.0 (29.2–35.0)
	(*e*)	3.9 (2.9–5.3)	4.1 (3.0–5.5)	14.0 (12.0–16.3)	14.1 (12.1–16.4)
2	(*a*)	6.7 (5.3–8.4)	6.6 (5.2–8.3)	29.1 (26.4–32.0)	38.3 (35.3–41.4)
	(*b*)	5.0 (3.8–6.5)	4.6 (3.5–6.1)	42.4 (39.4–45.5)	48.8 (45.7–51.9)
	(*c*)	5.0 (3.8–6.5)	5.8 (4.5–7.4)	44.6 (41.6–47.7)	48.9 (45.8–52.0)
	(*d*)	6.1 (4.8–7.8)	6.2 (4.9–7.9)	42.3 (39.3–45.4)	46.7 (43.6–49.8)
	(*e*)	5.9 (4.6–7.5)	6.2 (4.9–7.9)	19.5 (17.2–22.1)	19.0 (16.7–21.6)
3	(*a*)	5.2 (4.0–6.8)	5.0 (3.8–6.5)	90.4 (88.4–92.1)	92.3 (90.5–93.8)
	(*b*)	4.9 (3.7–6.4)	5.1 (3.9–6.6)	99.7 (99.1–99.9)	99.8 (99.3–100)
	(*c*)	6.3 (5.0–8.0)	6.2 (4.9–7.9)	99.8 (99.3–100)	99.8 (99.3–100)
	(*d*)	4.4 (3.3–5.9)	4.4 (3.3–5.9)	99.6 (99.0–99.8)	99.6 (99.0–99.8)
	(*e*)	4.4 (3.3–5.9)	4.4 (3.3–5.9)	72.9 (70.1–75.6)	72.9 (70.1–75.6)
4	(*a*)	6.4 (5.0–8.1)	5.7 (4.4–7.3)	10.2 (8.5–12.2)	19.4 (17.1–22.0)
	(*b*)	5.3 (4.1–6.9)	5.6 (4.3–7.2)	37.8 (34.9–40.9)	45.6 (42.5–48.7)
	(*c*)	5.7 (4.4–7.3)	4.9 (3.7–6.4)	72.2 (69.3–74.9)	74.9 (72.1–77.5)
	(*d*)	3.1 (2.2–4.4)	3.7 (2.7–5.1)	34.6 (31.7–37.6)	44.6 (41.6–47.7)
	(*e*)	4.5 (3.4–6.0)	4.2 (3.1–5.6)	9.7 (8.0–11.7)	15.7 (13.6–18.1)
5	(*a*)	4.3 (3.2–5.7)	4.3 (3.2–5.7)	29.9 (27.1–32.8)	29.9 (27.1–32.8)
	(*b*)	4.3 (3.2–5.7)	4.3 (3.2–5.7)	30.6 (27.8–33.5)	30.6 (27.8–33.5)
	(*c*)	6.9 (5.5–8.6)	6.9 (5.5–8.6)	14.5 (12.5–16.8)	14.5 (12.5–16.8)
6	(*a*)	3.3 (2.4–4.6)	3.3 (2.4–4.6)	92.6 (90.8–94.1)	92.6 (90.8–94.1)
	(*b*)	4.4 (3.3–5.9)	4.4 (3.3–5.9)	90.5 (88.5–92.2)	90.5 (88.5–92.2)
	(*c*)	4.4 (3.3–5.9)	4.4 (3.3–5.9)	53.7 (50.6–56.8)	53.7 (50.6–56.8)
7	(*a*)	5.6 (4.3–7.2)	5.5 (4.3–7.1)	11.0 (9.2–13.1)	8.8 (7.2–10.7)
	(*b*)	5.2 (4.0–6.8)	4.4 (3.3–5.9)	6.5 (5.1–8.2)	7.8 (6.3–9.6)
	(*c*)	5.7 (4.4–7.3)	4.8 (3.6–6.3)	5.8 (4.5–7.4)	6.9 (5.5–8.6)
8	(*a*)	4.6 (3.5–6.1)	4.5 (3.4–6.0)	78.7 (76.1–81.1)	78.1 (75.4–80.6)
	(*b*)	4.6 (3.5–6.1)	5.6 (4.3–7.2)	40.7 (37.7–43.8)	45.5 (42.4–48.6)
	(*c*)	4.7 (3.6–6.2)	4.8 (3.6–6.3)	11.6 (9.8–13.7)	19.3 (17.0–21.9)

**Table 7:** A summary of the results for the 1536 simulations with different parameters. The amount of error type I is calculated for the 768 simulations without signal (*β*_1_ = 0). Confidence intervals (CI) at 95% were computed around the nominal level *α* = 0.05, and the observed amount of errors for each regression scenario and for each method was compared with this interval. Methods that mostly remain within the CI are the most appropriate. Methods that frequently produce results below the interval are *conservative*; those above are *invalid*. Power was calculated for the remaining 768 simulations, which contained signal (*β*_1_ = 0.5).

Method	Proportion of error type i			Average power
	Within ci	Below ci	Above ci	
Draper–Stoneman	86.33%	8.20%	5.47%	72.96%
Still–White	67.84%	14.58%	17.58%	71.82%
Freedman–Lane	88.67%	8.46%	2.86%	73.09%
ter Braak	83.59%	11.07%	5.34%	73.38%
Kennedy	77.60%	1.04%	21.35%	74.81%
Manly	73.31%	15.89%	10.81%	73.38%
Smith	89.32%	7.81%	2.86%	72.90%
Huh–Jhun	85.81%	9.24%	4.95%	71.62%
Parametric	77.47%	14.84%	7.68%	72.73%

**Table 8:** Proportion of error type I (for *a* = 0.05), for some representative of the 768 simulation scenarios that did not have signal, using the different permutation methods, and with *G* as the statistic in the absence of EB (so, equivalent to the *F* statistic). Confidence intervals (95%) are shown in parenthesis.

Simulation parameters								Proportion of error type i (%)								
*N*	**x**_1_	**z**_1_	*ρ*		*ϵ*	ee	ise	D–S	S–W	F–L	tB	K	M	S	H–J	P
12	c	c	0	✗	N	✓	✗	4.9 (3.7–6.4)	5.3 (4.1–6.9)	5.1 (3.9–6.6)	5.3 (4.1–6.9)	5.3 (4.1–6.9)	5.0 (3.8–6.5)	4.9 (3.7–6.4)	4.7 (3.6–6.2)	4.4 (3.3–5.9)
12	c	c	0	✗	U	✓	✓	5.3 (4.1–6.9)	6.9 (5.5–8.6)	5.1 (3.9–6.6)	5.2 (4.0–6.8)	6.9 (5.5–8.6)	5.8 (4.5–7.4)	5.3 (4.1–6.9)	5.2 (4.0–6.8)	4.6 (3.5–6.1)
12	c	c	0	✗	W	✓	✗	5.9 (4.6–7.5)	6.5 (5.1–8.2)	5.2 (4.0–6.8)	5.4 (4.2–7.0)	6.5 (5.1–8.2)	5.0 (3.8–6.5)	5.9 (4.6–7.5)	5.4 (4.2–7.0)	8.3 (6.7–10.2)
12	c	c	0	✗	E	✓	✓	5.3 (4.1–6.9)	6.9 (5.5–8.6)	5.1 (3.9–6.6)	4.7 (3.6–6.2)	6.9 (5.5–8.6)	5.0 (3.8–6.5)	5.3 (4.1–6.9)	4.8 (3.6–6.3)	5.7 (4.4–7.3)
12	c	c	0.8	✗	N	✓	✗	4.4 (3.3–5.9)	3.6 (2.6–4.9)	5.1 (3.9–6.6)	5.2 (4.0–6.8)	5.8 (4.5–7.4)	4.8 (3.6–6.3)	5.1 (3.9–6.6)	4.4 (3.3–5.9)	4.4 (3.3–5.9)
12	c	c	0.8	✗	W	✓	✗	1.5 (0.9–2.5)	1.2 (0.7–2.1)	4.8 (3.6–6.3)	5.2 (4.0–6.8)	6.5 (5.1–8.2)	4.9 (3.7–6.4)	5.8 (4.5–7.4)	5.8 (4.5–7.4)	8.5 (6.9–10.4)
12	c	c	0.8	✗	N	✓	✓	5.5 (4.2–7.1)	5.4 (4.2–7.0)	4.9 (3.7–6.4)	5.4 (4.2–7.0)	7.5 (6.0–9.3)	4.8 (3.6–6.3)	4.8 (3.6–6.3)	5.8 (4.5–7.4)	4.6 (3.5–6.1)
12	c	c	0.8	✓	N	✓	✓	5.1 (3.9–6.6)	7.2 (5.8–9.0)	5.4 (4.2–7.0)	4.3 (3.2–5.7)	7.2 (5.8–9.0)	5.2 (4.0–6.8)	5.1 (3.9–6.6)	4.6 (3.5–6.1)	4.6 (3.5–6.1)
12	c	d	0	✗	W	✓	✗	5.6 (4.3–7.2)	6.8 (5.4–8.5)	5.4 (4.2–7.0)	4.7 (3.6–6.2)	6.8 (5.4–8.5)	4.0 (3.0–5.4)	5.6 (4.3–7.2)	3.7 (2.7–5.1)	8.9 (7.3–10.8)
12	c	d	0	✗	N	✓	✗	3.9 (2.9–5.3)	4.9 (3.7–6.4)	3.9 (2.9–5.3)	4.0 (3.0–5.4)	4.9 (3.7–6.4)	4.3 (3.2–5.7)	3.9 (2.9–5.3)	4.2 (3.1–5.6)	3.7 (2.7–5.1)
12	c	d	0	✗	W	✗	✓	2.9 (2.0–4.1)	4.3 (3.2–5.7)	2.6 (1.8–3.8)	2.8 (1.9–4.0)	4.3 (3.2–5.7)	14.1 (12.1–16.4)	2.9 (2.0–4.1)	16.4 (14.2–18.8)	9.0 (7.4–10.9)
12	d	d	0	✗	W	✓	✗	3.2 (2.3–4.5)	4.6 (3.5–6.1)	2.2 (1.5–3.3)	2.0 (1.3–3.1)	4.6 (3.5–6.1)	3.8 (2.8–5.2)	3.2 (2.3–4.5)	2.6 (1.8–3.8)	0.5 (0.2–1.2)
24	c	c	0.8	✗	N	✓	✗	4.4 (3.3–5.9)	3.5 (2.5–4.8)	4.3 (3.2–5.7)	4.4 (3.3–5.9)	4.9 (3.7–6.4)	4.4 (3.3–5.9)	4.3 (3.2–5.7)	4.5 (3.4–6.0)	4.4 (3.3–5.9)
24	d	d	0	✗	N	✓	✗	5.0 (3.8–6.5)	5.4 (4.2–7.0)	5.1 (3.9–6.6)	5.1 (3.9–6.6)	5.4 (4.2–7.0)	4.9 (3.7–6.4)	5.0 (3.8–6.5)	4.5 (3.4–6.0)	5.0 (3.8–6.5)
24	d	d	0	✗	U	✓	✗	6.2 (4.9–7.9)	6.6 (5.2–8.3)	6.3 (5.0–8.0)	5.9 (4.6–7.5)	6.6 (5.2–8.3)	5.5 (4.2–7.1)	6.2 (4.9–7.9)	5.9 (4.6–7.5)	5.8 (4.5–7.4)
24	d	d	0.8	✗	U	✓	✗	4.9 (3.7–6.4)	1.8 (1.1–2.8)	5.1 (3.9–6.6)	4.8 (3.6–6.3)	5.4 (4.2–7.0)	5.1 (3.9–6.6)	5.2 (4.0–6.8)	5.7 (4.4–7.3)	5.4 (4.2–7.0)
48	c	c	0	✗	N	✗	✓	4.9 (3.7–6.4)	5.4 (4.2–7.0)	5.0 (3.8–6.5)	5.6 (4.3–7.2)	5.4 (4.2–7.0)	3.8 (2.8–5.2)	4.9 (3.7–6.4)	6.0 (4.7–7.6)	5.0 (3.8–6.5)
48	c	c	0.8	✓	U	✓	✗	5.1 (3.9–6.6)	5.4 (4.2–7.0)	5.0 (3.8–6.5)	5.7 (4.4–7.3)	5.4 (4.2–7.0)	5.2 (4.0–6.8)	5.1 (3.9–6.6)	5.6 (4.3–7.2)	5.6 (4.3–7.2)
48	c	c	0.8	✓	N	✓	✗	4.6 (3.5–6.1)	4.8 (3.6–6.3)	4.7 (3.6–6.2)	4.7 (3.6–6.2)	4.8 (3.6–6.3)	4.6 (3.5–6.1)	4.6 (3.5–6.1)	4.4 (3.3–5.9)	4.5 (3.4–6.0)
48	c	d	0	✗	E	✗	✓	5.4 (4.2–7.0)	5.7 (4.4–7.3)	5.1 (3.9–6.6)	5.5 (4.2–7.1)	5.7 (4.4–7.3)	9.2 (7.6–11.2)	5.4 (4.2–7.0)	4.3 (3.2–5.7)	5.1 (3.9–6.6)
48	c	d	0.8	✗	E	✓	✗	5.5 (4.2–7.1)	0.3 (0.1–0.9)	5.0 (3.8–6.5)	5.0 (3.8–6.5)	5.0 (3.8–6.5)	4.9 (3.7–6.4)	5.0 (3.8–6.5)	5.0 (3.8–6.5)	4.9 (3.7–6.4)
96	c	c	0	✗	N	✓	✓	5.1 (3.9–6.6)	5.3 (4.1–6.9)	5.1 (3.9–6.6)	4.9 (3.7–6.4)	5.3 (4.1–6.9)	4.6 (3.5–6.1)	5.1 (3.9–6.6)	5.3 (4.1–6.9)	4.9 (3.7–6.4)
96	c	c	0.8	✗	N	✗	✓	5.0 (3.8–6.5)	3.6 (2.6–4.9)	5.0 (3.8–6.5)	4.8 (3.6–6.3)	5.2 (4.0–6.8)	4.4 (3.3–5.9)	5.1 (3.9–6.6)	5.2 (4.0–6.8)	4.9 (3.7–6.4)
96	d	c	0	✗	W	✓	✗	4.9 (3.7–6.4)	5.2 (4.0–6.8)	4.7 (3.6–6.2)	4.8 (3.6–6.3)	5.2 (4.0–6.8)	4.5 (3.4–6.0)	4.9 (3.7–6.4)	3.9 (2.9–5.3)	3.6 (2.6–4.9)

*N*: number of observations; **x**_1_ and **z**_1_: regressors of interest and of no interest, respectively, being either continuous (c) or discrete (d). *ρ*: correlation between **x**_1_ and **z**_1_; : model partitioned or not (using the scheme of Beckmann et al. (2001), shown in Appendix A"); ϵ: distribution of the simulated errors, which can be normal (N), uniform (U), exponential (E) or Weibull (W); ee: errors treated as exchangeable; ise: errors treated as independent and symmetric. The methods are the same shown in Table 2: Draper–Stoneman (D–S), Still–White (S–W), Freedman–Lane (F–L), ter Braak (tB), Kennedy (K), Manly (M), Huh–Jhun (H–J), Smith (S) and parametric (P), the last not using permutations.

## Appendix A Model partitioning

The permutation methods discussed in this paper require that the design matrix **M** is partitioned into effects of interest and nuisance effects. Such partitioning is not unique, and schemes can be as simple as separating apart the columns of **M** as [**X Z**], with *ψ* = [*β*′ *γ*′]  (Guttman, 1982). More involved strategies can, however, be devised to obtain some practical benefits. One such partitioning is to define **X** = **MDC**(**C**′**DC**)^− 1^ and **Z** = **MDC**_*v*_(**C**_*v*_′**DC**_*v*_)^− 1^, where **D** = (**M**′**M**)^− 1^, **C**_*v*_ = **C**_*u*_ − **C**(**C**′**DC**)^− 1^**C**′**DC**_*u*_, and **C**_*u*_ has *r* − rank(**C**) columns that span the null space of **C**, such that [**C C**_*u*_] is a *r* × *r* invertible, full-rank matrix (Beckmann et al., 2001; Smith et al., 2007). This partitioning has a number of features: $\hat{\beta }=C^{\prime }\hat{\psi }$, $\hat{\mathrm{Cov}}\left(\hat{\beta }\right)=C^{\prime }\hat{\mathrm{Cov}}\left(\hat{\psi }\right)C$, i.e., estimates and variances of *β* for inference on the partitioned model correspond exactly to the same inference on the original model, **X** is orthogonal to **Z**, and span(**X**) ∪ span(**Z**) = span(**M**), i.e., the partitioned model spans the same space as the original. This is the partitioning strategy used in this paper, and used in randomise (see Appendix B).

Another partitioning scheme, derived by Ridgway (2009), defines **X** = **M**(**C**^+^)′ and **Z** = **M** − **MCC**^+^. As with the previous strategy, the parameters of interest in the partitioned model are equal to the contrast of the original parameters. A full column rank nuisance partition can be obtained from the singular value decomposition (svd) of **Z**, which will also provide orthonormal columns for the nuisance partition. Orthogonality between regressors of interest and nuisance can be obtained by redefining the regressors of interest as **R_Z_X**.

## Appendix B The randomise algorithm

Algorithm 1 describes a procedure for permutation inference on contrasts of the glm parameter estimates using the Freedman–Lane method. Modifications for other methods are trivial. For this algorithm, consider **Y** as a four-dimensional array, being the first three dimensions for space and the last for an observation index. A variable **v** = [*x*, *y*, *z*] is used to specify the point position in space, so that the vector of *N* different observations per point is represented as **Y**[**v**]. A set C of contrasts is specified, as well as the unpartitioned design matrix **M**. Indicator variables are used to specify whether the errors should be treated as exchangeable (ee = true), independent and symmetric (ise = true), or both, which allows for permutations to happen together with sign flipping. A positive integer *J* is specified as the number permutations to be performed. Optionally, a *N* × 1 vector **b** is provided to indicate the *B* exchangeability blocks that group the observations, along with an indicator variable PB that informs whether blocks should be permuted as a whole (pb = true), or if permutations should happen within block only (pb = false). The specification of **b** and pb obviate the need to specify the variance groups, as these can be defined implicitly for within or whole-block permutation when the pivotal statistic is computed. Algorithm 1 The randomise algorithm

In the algorithm, the statistics *T* for each point (voxel, vertex, face) are stored in the array **T**, whereas the counters are stored in the arrays **U** and **F**. The design matrix as well as the contrasts can be specific for each image point (voxelwise, vertexwise, facewise), and there is no challenge other than implementation. It is possible to omit the for-loop between lines 57 and 61, and instead store the distribution of the largest statistic as a vector of size *J*, which is then used to assess significance. The code runs faster, but it would be slightly less clear to present. In programming languages that offer good matrix manipulation capabilities, e.g. Octave, matlab or r, the for-loops that iterate for each point **v** can be replaced by matrix operations that are executed all in a single step. In the fmrib Software Library (fsl),^6^ a fast implementation, in C++, of the randomise algorithm is available.

## Appendix C Worked examples

The examples below serve to illustrate the permutation aspects discussed in the paper, all with tiny samples, *N* = 12 only, so that the design matrices can be shown in their full extent. While permutation tests in general remain valid even with such small samples, these examples are by no means to be understood as a recommendation for sample sizes. There are many reasons why larger samples are more appropriate (see Button et al. (2013) for a recent review), and in what concerns permutation methods, larger samples allow smaller p-values, improve the variance estimates for each vg (which are embodied in the weighting matrix under restricted exchangeability), and allow finer control over the familywise error rate. For each example, the relevant contrasts are also shown. Example 1 Mean effect Consider a multi-subject fmri study to investigate the bold response associated with a novel experimental task. After the first-level analysis (within subject), maps of contrasts of parameter estimates for each subject are used in a second level analysis. The regressor for the effect of interest (the mean effect) is simply a column of ones; nuisance variables, such as handedness, can be included in the model. Permutations of the data or of the design matrix do not change the model with respect to the regressor of interest. However, by treating the errors as symmetric, instead of permutation, the signs of the ones in the design matrix, or of each datapoint, can be flipped randomly to create the empirical distribution from which inference can be performed. The procedure can be performed as in either the Freedman–Lane or Smith methods (Table 9). Example 2 Multiple regression Consider the analysis of a study that compares patients and controls with respect to brain cortical thickness, and that recruiting process ensured that all selected subjects are exchangeable. Elder subjects may, however, have thinner cortices, regardless of the diagnosis. To control for the confounding effect of age, it is included in the design as a nuisance regressor. Sex is also included. The permutation strategy follows the Freedman–Lane or Smith methods, with the residuals of the reduced model being permuted under unrestricted exchangeability (Table 10). Example 3 Paired *t*-test Consider a study to investigate the effect of the use of a certain analgesic in the magnitude of the bold response associated with painful stimulation. In this example, the response after the treatment is compared with the response before the treatment, i.e., each subject is their own control. The experimental design is the “paired *t*-test”. One eb is defined per subject, as the observations are not exchangeable freely across subjects, and must remain together in all permutations. In this example, in the absence of evidence on the contrary, the variance was assumed to be homogeneous across all observations, such that only one vg, encompassing all, was defined (Table 11). If instead of just two, there were more observations per subject being compared, the same strategy, with the necessary modifications to the design matrix, could be applied only under the assumption of compound symmetry, something clearly invalid for most studies, albeit not for all. Some designs with repeated measurements can, however, bypass this need altogether, as shown in Example 6. Example 4 Unequal group variances Consider a study using fmri to compare whether the bold response associated with a certain cognitive task would differ among subjects with autistic spectrum disorder (asd) and control subjects, while taking into account differences in age and sex. In this hypothetical example, the cognitive task is known to produce more erratic signal changes in the patient group than in controls. Therefore, variances cannot be assumed to be homogeneous with respect to the group assignment of subjects. This is an example of the classical Behrens–Fisher problem. To accommodate heteroscedasticity, two permutation blocks are defined according to the group of subjects. Under the assumption of independent and symmetric errors, the problem is solved by means of random sign flipping (Pesarin, 1995), using the well known Welch's *v* statistic, a particular case of the statistic *G* shown in Eq. (6) (Table 12). Example 5 Variance as a confound Consider a study using fmri to compare whether a given medication would modify the bold response associated with a certain attention task. The subjects are allocated in two groups, one receiving the drug, the other not. In this hypothetical example, the task is known to produce very robust and, on average, similar responses for male and female subjects, although it is also known that males tend to display more erratic signal changes, either very strong or very weak, regardless of the drug. Therefore, variances cannot be assumed to be homogeneous with respect to the sex of the subjects. To accommodate heteroscedasticity, two permutation blocks are defined according to sex, and each permutation matrix is constructed such that permutations only happen within each of these blocks (Table 13). Example 6 Longitudinal study Consider a study to evaluate whether fractional anisoptropy (fa) would mature differently between boys and girls during middle childhood. Each child recruited to the study is examined three times, at the ages of 9, 10 and 11 years, and none of them are related in any known way. Permutation of observations within child cannot be considered, as the null hypothesis is not that fa itself would be zero, nor that there would be no changes in the value of fa along the three yearly observations, but that there would be no difference in potential changes between the two groups; the permutations must, therefore, always keep in the same order the three observations. Moreover, with three observations, it might be untenable to suppose that the joint distribution between the first and second observations would be the same as for between the first and third, even though it might be the same as for the second and third; if these three pairwise joint distributions cannot be assumed to be the same, this precludes within-block exchangeability. Instead, blocks are defined as one per subject, each encompassing all the three observations, and permutation of each block as a whole is performed. It is still necessary, however, that the covariance structure within block (subject) is the same for all blocks, preserving exchangeability. If the variances cannot be assumed to be identical along time, one variance group can be defined per time point, otherwise all are assigned to the same vg (as in Example 3). If there are nuisance variables to be considered (some measurements of nutritional status, for instance), these can be included in the model and the procedure is performed using the same Freedman–Lane or Smith strategies (Table 14).

## Footnotes

1 While we focus on univariate data, the general principles presented can be applied to multivariate linear models.

2 We name this method after Smith because, although orthogonalisation is a well known procedure, it does not seem to have been proposed by anyone to address the issues with permutation methods with the glm until Smith and others presented it in a conference poster (Nichols et al., 2008). We also use the eponym to keep it consistent with Ridgway (2009),and to keep the convention of calling the methods by the earliest author that we could identify as the proponent for each method, even though this method seems to have been proposed by an anonymous referee of O'Gorman (2005).

3 Note that, for clarity, *G* is defined in Eq. (6) as a function of **M**, *ψ* and **C** in the unpartitioned model. With the partitioning described in the Appendix A, each of these variables is replaced by their equivalents in the partitioned, full model, i.e., [**X Z**], [*β*′ *γ*′]′ and [I_*s* × *s*_ 0_*s* × (*r* − *s*)_]′ respectively.

4 If the errors are independent and normally distributed, yet not necessarily with equal variances (i.e., Λ ≠ 1), parametric p-values for *G* can be approximated by referring to the *F*-distribution with degrees of freedom *v*_1_ = *s* and *v*_2_ = 2(*s* − 1)/3/(Λ − 1).

5 Parametric p-values for these two statistics, however, differ. If computed, parametric p-values would have to consider that the degrees of freedom for the *G* statistic are not the same as for *F*; see footnote 4.
